# High-Performing Vacuum-Pressure Swing Adsorption Unit for Drying Hydrogen from Water Electrolysis

**DOI:** 10.1021/acs.iecr.6c00750

**Published:** 2026-08-04

**Authors:** Tiago M. R. Santos, José M. Sousa, Frederico Relvas, Adélio Mendes

**Affiliations:** † LEPABE - Laboratory for Process Engineering, Environment, Biotechnology and Energy, Faculty of Engineering, 112048 University of Porto, Rua Dr. Roberto Frias, Porto 4200-465, Portugal; ‡ ALiCE − Associate Laboratory in Chemical Engineering, Faculty of Engineering, University of Porto, Rua Dr. Roberto Frias, Porto 4200-465, Portugal; § Amnis Pura, Parque Empresarial Laborim, 100, Armazém C3, Vila Nova de Gaia 4430-246, Portugal; ∥ Departamento de Química − Escola de Ciências da Vida E Do Ambiente, Universidade de Trás-os-Montes e Alto Douro, Vila-Real 5001-911, Portugal

## Abstract

The growing demand for ultrapure hydrogen in fuel cell applications requires the development of efficient and cost-effective purification technologies. This study reports the development, optimization, and techno-economic assessment of a vacuum-pressure swing adsorption (VPSA) system for hydrogen drying in compliance with the ISO 14687-2 standard. Activated alumina was employed as the adsorbent, and experimental validation demonstrated a reduction in water content to dew points below −65.5 °C, with a concentration of 5 μmol of H_2_O per mol of H_2_. Adsorption isotherms for hydrogen and water were measured and modeled using the Langmuir and Toth-Aranovich-Donohue equations. Several regeneration strategies were evaluated, with the combined application of vacuum and purge gas identified as the most effective. A Box-Behnken response surface methodology was employed to optimize operating parameters, and the optimal conditions were experimentally validated. Under these conditions, the VPSA achieved a hydrogen recovery of 98.98%, a productivity of 3.92 kg_H_2__·kg_ads_^–1^·cycle^–1^, and a dew point of −66.76 °C. A techno-economic analysis of a hypothetical unit producing 300 kg·h^–1^ of hydrogen, scaled from laboratory data, demonstrated the economic feasibility of the VPSA process. When hydrogen was fed in a compressed state at 7.34 bar, the additional specific energy consumption and cost associated with the purification step were estimated at 0.038 kWh·kg^–1^ and 0.039 €·kg^–1^ of H_2_, respectively. These results highlight the VPSA system as a promising solution for high-purity hydrogen drying, combining robust operational performance with strong economic viability.

## Graphical abstract

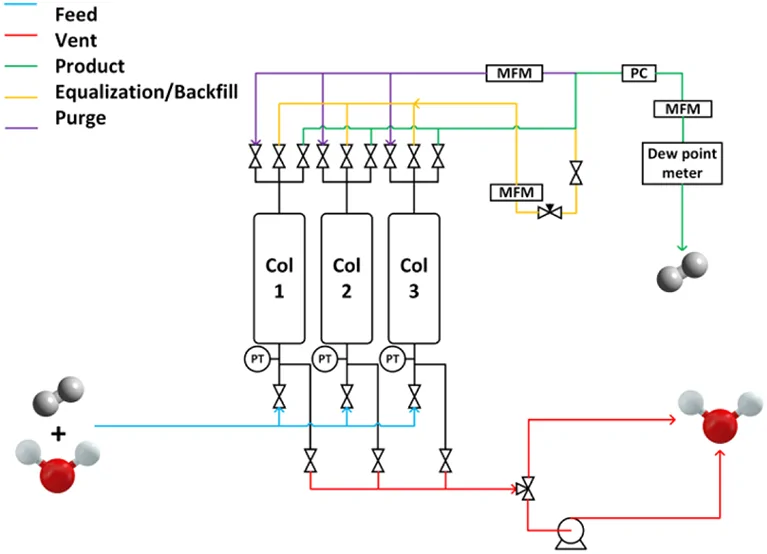

## Introduction

Global warming remains a critical challenge that requires rapid socioeconomic adaptation. Rising global temperatures and the increasing frequency of extreme climate events continue to trigger humanitarian crises and economic disruptions. Such events force an increase in energy production and expenditure that consequently emit additional greenhouse gases creating a potentially irreversible snowball effect.^1^ Despite predictions for the oil and natural gas demand to peak within the current decade,^2^ global CO_2_ emissions reached 53.2 Gt in 2024, a 1.3% increase from the previous year.^3^ The latest data from 2022 reveals that electricity and heat sector accounts for 32.9% of emissions and rising steadily since the past decades^4^ which can be partly attributed to harsher environmental conditions. Fossil fuels remain the dominant contributors, with coal, oil, and natural gas accounting for 40.9%, 32.3%, and 20.8% of emissions, respectively as of 2024.^5^ The ongoing energy crisis has accelerated demand for renewable technologies^2^ and in this context, the International Energy Agency reports that investment in the energy sector worldwide will have risen 2% in 2025, up to 3.3 trillion USD, from the previous year where two-thirds account for clean energy sectors.^6^ The European Union is expected to have invested 386.31 billion USD in 2025, a 5.8% increase from the previous year.^6^

In addition to the electricity and heat sector, the transport sector is the second highest producer of CO_2_ emissions with a 16.1% contribution.^4^ Green hydrogen has a significant potential for mitigation of emissions across both sectors since it can serve as a fuel, energy carrier, or a feedstock for chemical synthesis.^7^ Hydrogen is a nontoxic, highly combustible gas that can be produced from renewable resources. Its combustion generates only water vapor, releasing 120 MJ·kg^–1^ (lower heating value),^8^ nearly three times the mass energy density of gasoline or diesel.^9^ Even so, more than 99% of global hydrogen production currently relies on fossil-based routes, primarily steam methane reforming and coal gasification, which collectively emitted approximately 920 Mt of CO_2_ in 2023.^10,11^ Transitioning to low-carbon hydrogen represents a substantial contribution to reduce global emissions. However, unless consumed on-site, hydrogen requires safe and scalable storage and transport solutions.

Compressed gas storage is the most mature technology for hydrogen storage. Type I vessels, rated for 150–300 bar, are mainly used in stationary applications due to their low volumetric energy density. Type II vessels consist of seamless steel cylinders reinforced with carbon fiber and can withstand pressures up to 1000 bar, making them suitable for hydrogen refueling stations. Lighter and fully composite Type III and Type IV vessels, with and without a metal liner, respectively, operate at up to 700 bar, offering higher volumetric energy density than type I, rendering them the preferred option for mobility applications.^11,12^

Green hydrogen can be produced via water electrolysis powered by renewable electricity.^13,14^ Its purity depends on the type of electrolyzer, namely alkaline or anion exchange membrane (AEM), which influences the purity due to the presence of water at the cathode;^15^ proton exchange membrane (PEM) influences the purity due to crossover of water and dissolved gases^16^ as well as ion crossover through the separator.^17^ The quality of the raw materials also influences purity.^18^

Hydrogen purity requirements vary by application and are defined by international standards. ISO 14687-2 specifies limits for 13 common contaminants in hydrogen intended for fuel cell use. According to Bacquardt,^15^ PEM electrolysis can introduce nitrogen, oxygen, and water as the main cathodic impurities. Nitrogen, typically used for inertisation, can be purged during startup until levels drop below 100 μmol·mol^–1^ of H_2_. Oxygen and water, arising from crossover phenomena, must be maintained below 5 μmol·mol^–1^ of H_2_ to avoid risks of explosive mixtures and condensation in Type III and IV storage vessels. Oxygen is typically removed with a DeOxo unit, converting it to water via catalytic reduction over Pd-based catalysts, albeit at the expense of hydrogen yield.^19^

Cyclic adsorption-based purification provides the most favorable trade-off between costs, hydrogen product quality, and recovery, with drying performance strongly dependent on the regeneration strategy.^20^ Pressure swing adsorption (PSA) is a widely implemented technique that operates with adsorption at elevated pressure and regeneration at atmospheric pressure. Variants include vacuum-pressure swing adsorption (VPSA)^21^ and vacuum swing adsorption (VSA),^22^ which use regeneration pressures below atmospheric pressure, and temperature swing adsorption (TSA),^23^ which relies on thermal regeneration at the expense of higher energy consumption and longer cycle times. Other technologies can be employed for hydrogen purification, such as membrane separation or metal hydride adsorption, but they present technical limitations, such as the driving force for water permeation that becomes too thin for low dew point temperatures, making the membrane-based purification very challenging; on the other hand, metal hydrides are high cost and low durability and achieve low recovery.^24^

Gas separation in adsorbents may occur through equilibrium or kinetic mechanisms.^20^ For instance, nitrogen separation from air using carbon molecular sieve adsorbents relies on kinetic selectivity, because N_2_ diffuses slower, whereas zeolites are employed for oxygen purification under equilibrium conditions owing to their stronger affinity for N_2_. Conventional adsorption dryers operate under quasi-adsorption equilibrium conditions and typically use a two-column system loaded with silica gel, activated alumina, or zeolite molecular sieves. Silica gel is most effective at high relative humidity (RH > 50%)^25^ due to multilayer water adsorption within its meso- and macroporous structure.^26,27^ Zeolites exhibit superior performance at low RH (<30%) because of strong water-framework interactions, though rapid saturation occurs due to limited pore volume.^28,29^ Activated alumina offers intermediate behavior, combining strong adsorption sites at low RH with multilayer adsorption enabled by its meso- and macroporosity.^30,31^ Other adsorbents include activated carbon, which commonly exhibits characteristics like silica gel, with predominantly meso- and macroporous adsorption,^32^ and metal–organic frameworks (MOFs), which are engineered to maximize water adsorption within well-defined crystalline structures, analogous to zeolites. However, MOFs are still in relatively early stages of development and currently exhibit limited commercial applicability.^33^

Despite the growing relevance of green hydrogen, relatively few studies have addressed equilibrium adsorption-based purification strategies. Ligen et al.^34^ reported a pilot-scale VPSA system containing zeolite 4A packed beds integrated with a DeOxo, achieving a hydrogen recovery of 98.4% at 35 to 38 bar with an inlet water concentration of 1000 ppm and an energy consumption of 0.5 kWh·kg^–1^ of H_2_. Tjarks et al.^35^ modeled a two-column TSA system operating at 10 bar with an inlet water vapor concentration of 44 354 ppm using zeolite 13X. The authors predict improved efficiency at elevated pressures and report an energy consumption of 0.167 kWh·kg^–1^ of H_2_, although experimental validation was not provided. Table 1 summarizes the key findings of both authors.

**Table 1:** Comparison between Existing Green Hydrogen Purification Publications

Author	Unit	Adsorbent	Inlet H_2_O/ppm	Pressure/bar	H_2_ recovery	Energy consumption/kWh·kg^–1^
Ligen et al.	VPSA	4A zeolite	1000	35–38	98.4%	0.5
Tjarks et al.	TSA	13X zeolite	44 354	10	-	0.167

This work investigates the techno-economic feasibility of drying humid hydrogen streams, with particular emphasis on the underreported influence of operating conditions on the performance of green hydrogen adsorption dryers. The adsorption capacity of a commercial activated alumina was experimentally determined, and adsorption dynamics were obtained from breakthrough experiments. A laboratory-scale VPSA unit was optimized using response surface methodology to achieve a target dew point of −65.5 °C (<5 μmol·mol^–1^ of H_2_) while maximizing hydrogen recovery. The experimental findings were subsequently used to perform a techno-economic assessment of a scaled-up VPSA system processing 300 kg_H_2__·h^–1^ of hydrogen, including evaluation of CAPEX, OPEX, and the corresponding levelized cost of hydrogen drying (LCOHD) at fuel-cell-grade purity.

## Experimental Section

### Characterization Unit and Methodology

Adsorption equilibrium isotherms for hydrogen and water vapor were determined on commercially available Induceramic activated alumina. The experimental setup used for these measurements is shown schematically in Figure S1. It is an in-house-built apparatus that applies the volumetric method.^36^ The procedure for the measurement of a single isotherm point of water and hydrogen is described in the Supporting Information.

For water vapor isotherms, the tank containing water and water vapor in equilibrium was evacuated before each measured isotherm to mitigate any dilution from undesired potential air leaks ensuring that only water vapor was loaded into the sample, thereby minimizing contamination from external gases. Prior to all isotherm experiments, the activated alumina adsorbent was regenerated overnight under a pure nitrogen flow at 375 °C.

### Adsorption Isotherm Modeling

The adsorption isotherms of hydrogen and water were investigated at 20 °C, 30 °C, and 40 °C. The experimental adsorption isotherms were fitted to the Langmuir equation for hydrogen—eq 1, and to the Toth-Aranovich-Donohue (Toth-AD)^37^ for water vapor—eq 2.

1 $$q_{H_{2}}=\left[\frac{q_{m}b_{1}P}{1+b_{1}P}\right]$$

2 $$q_{H_{2}O}=\left[\frac{q_{m}b_{2}P}{{\left(1+{\left(b_{2}P\right)}^{f}\right)}^{1/f}}\right]\left[\frac{1}{{\left(1-b_{3}P\right)}^{n}}\right]$$

where *q* is the adsorption capacity at a given equilibrium pressure *P*, *q*_*m*_ is the monolayer adsorption capacity, *b*_1_ is the Langmuir equilibrium constant at a given temperature, *b*_2_ is the Toth equilibrium constant at a given temperature, *b*_3_ is the Aranovich-Donohue equilibrium constant at a given temperature, *f* is a dimensionless heterogeneity parameter from the Toth factor, and *n* is a dimensionless fitting parameter from the Aranovich-Donohue factor.

The Langmuir equation assumes that the adsorbent surface is homogeneous and that adsorption proceeds only to a monolayer,^38^ an assumption consistent with the weak interactions exhibited by hydrogen under standard conditions, as it is a lightly polarizable molecule with limited affinity for both adsorbent surfaces and itself.^37^ Conversely, the Toth-AD model expands the Toth model, which assumes a heterogeneous interaction between the gas species and adsorbent in a monolayer^38^ represented by *f*, to the Aranovich-Donohue model, which accounts for the formation of multilayers,^37^ represented by *n*.

As mentioned, the equilibrium constants are a function of temperature according to eq 3.

3 $$b_{i}=b_{i}^{0}\exp\left[\frac{Q_{i}}{RT^{0}}\left(\frac{T^{0}}{T}-1\right)\right]$$

where *b*_i_ represents *b*_1_, *b*_2_ and *b*_3_ from eqs 1 and 2, $b_{i}^{0}$ is the temperature independent equilibrium constant of adsorption, R is the gas constant, *T* ^0^ is a reference temperature set to 273.15 K, *T* is the temperature of the isotherm, and *Q*_i_ is the adsorption heat. The heterogeneity parameter *f* is also dependent on temperature as given by eq 4.^38^

4 $$f=f^{0}+\alpha \left[1-\left(T^{0}/T\right)\right]$$

where *f* ^0^ is the temperature-independent heterogeneity factor, and *α* is the heterogeneity constant parameter. The physical validation of the Toth-AD equation was conducted by considering the physical meaning of the adsorption heats associated with both equilibrium constants. The Clausius–Clapeyron equation (eq 5) was used to calculate the temperature-independent isosteres

5 $${\frac{\partial \ln P}{\partial T}|}_{q}=\frac{Q}{RT^{2}}$$

where *∂* ln *P*/∂*T* is the rate of change of equilibrium pressure with temperature at constant loading. When integrated, eq 5 yields^39^

6 $$\ln P{|}_{q}=-\left(Q/RT\right)+C$$

The slope returns −Q/R when In *P* is plotted against 1/*T* at constant loadings, after which it is possible to relate heat of adsorption against the loading. *C* is an integration constant that, in absolute terms, represents the heat of vaporization.

### Breakthrough Characterization Unit and Methodology

The setup used for obtaining the breakthrough curves is sketched in Figure 1. It was built in-house, and described elsewhere.^31^ A bubbling column, W, filled with water, is used to humidify the inlet stream of hydrogen. The dew point temperature, *T*_dp_, of this stream is read at atmospheric conditions using a capacitive dew point meter (CS-Instruments FA500). Humidity is controlled by balancing the humidified and dry hydrogen inlets. The Magnus-Tetens equation^40^ was used to convert *T*_dp_ measured by the DPM to water vapor pressure, $P_{H_{2}O}$ , in eq 7.

7 $$P_{H_{2}O}=6.1078\times \exp\left(17.269388\times \frac{\left(T_{\mathrm{dp}}-273.15\right)}{\left(T_{\mathrm{dp}}-35.86\right)}\right)$$

**Figure 1:**
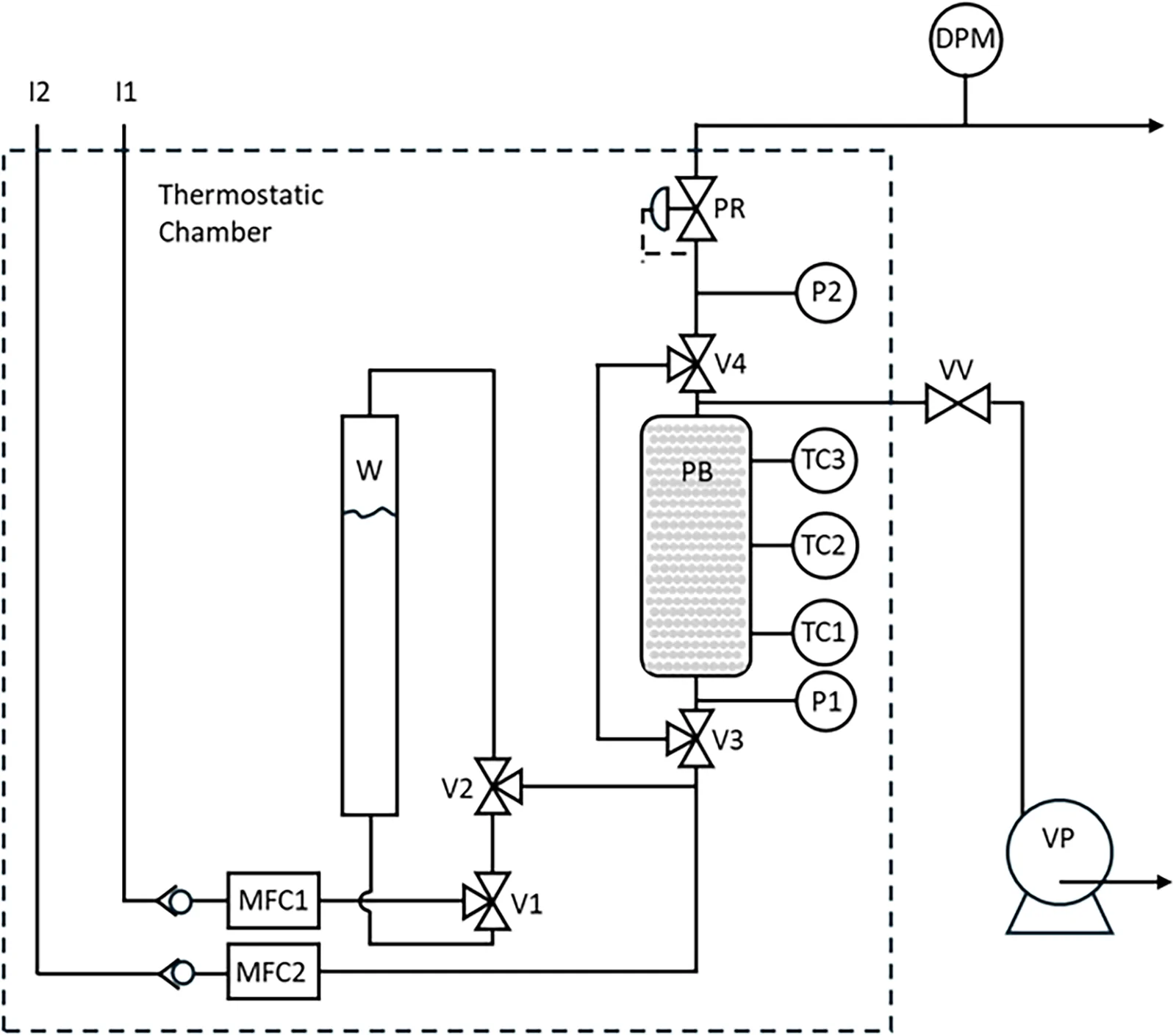
Breakthrough characterization unit. DPM: dew point meter; I1–I2: hydrogen inlets; MFC: mass flow controller; P1–P2: pressure transducers; PR: pressure regulator; TC1–TC3: thermocouples; V1–V4: three-way valve; VP: vacuum pump; VV: vacuum valve; W: water column.

The relative humidity is then calculated following $P_{H_{2}O}/P_{H_{2}O,s}$ , where $P_{H_{2}O,s}$ is the saturation pressure of water at a given temperature. The corresponding breakthrough curves for different temperatures, feed flow rates and RH were obtained. From the breakthrough curves, it is possible to determine the total bed adsorption capacity *q*_s_, according to eq 8:

8 $$q_{s}=\frac{F_{\mathrm{feed}}W_{0}}{60\times m_{\mathrm{ads}}}A_{s}=\frac{F_{\mathrm{feed}}W_{0}}{60\times m_{\mathrm{ads}}}\int_{0}^{t_{s}}\left(1-\frac{W_{s}}{W_{0}}\right)dt$$

where *F*_feed_ is the volumetric feed flow rate, *W*_0_ is the feedwater vapor concentration, *A*_s_ is the plotted area above the breakthrough curve, *m*_ads_ is the mass of adsorbent inside the column, *W*_*s*_ is the outlet water vapor concentration at time *t*, and *t*_*s*_ is the saturation time. All variables except *W*_s_ and *t*_s_ are known experimental parameters. The capacity at breakthrough time, *q*_b_, was obtained from the area above the breakthrough curve, according with eq 9:

9 $$q_{b}=\frac{F_{\mathrm{feed}}W_{0}}{60\times m_{\mathrm{ads}}}A_{b}=\frac{F_{\mathrm{feed}}W_{0}}{60\times m_{\mathrm{ads}}}\int_{0}^{t_{b}}\left(1-\frac{W_{b}}{W_{0}}\right)dt$$

where *A*_b_ is the plotted area above the breakthrough curve up to the experimental breakpoint concentration of 5 μmol·mol^–1^ of H_2_, and *t*_b_ is the breakpoint time. The height of the mass transfer zone (MTZ), *H*_MTZ_ is calculated from eq 10:

10 $$H_{\mathrm{MTZ}}=H_{\mathrm{bed}}\left[1-\left(q_{b}/q_{s}\right)\right]$$

where *H*_bed_ is the height of the packed bed. The efficiency of the packed bed (PB), *η*_ads_, is calculated with eq 11:

11 $$\eta_{\mathrm{ads}}=q_{b}/q_{s}\times 100$$

Table S1 (Supporting Information) summarizes the characteristic dimensions of the packed bed, and the experimental testing conditions.

### Laboratory VPSA Experiments

For running the VPSA experiments, an existing in-house-made test bench was modified and used.^41^ A three-column VPSA unit, designed to dry hydrogen streams, was constructed as sketched in Figure 2. The unit is described in detail in the Supporting Information.

**Figure 2:**
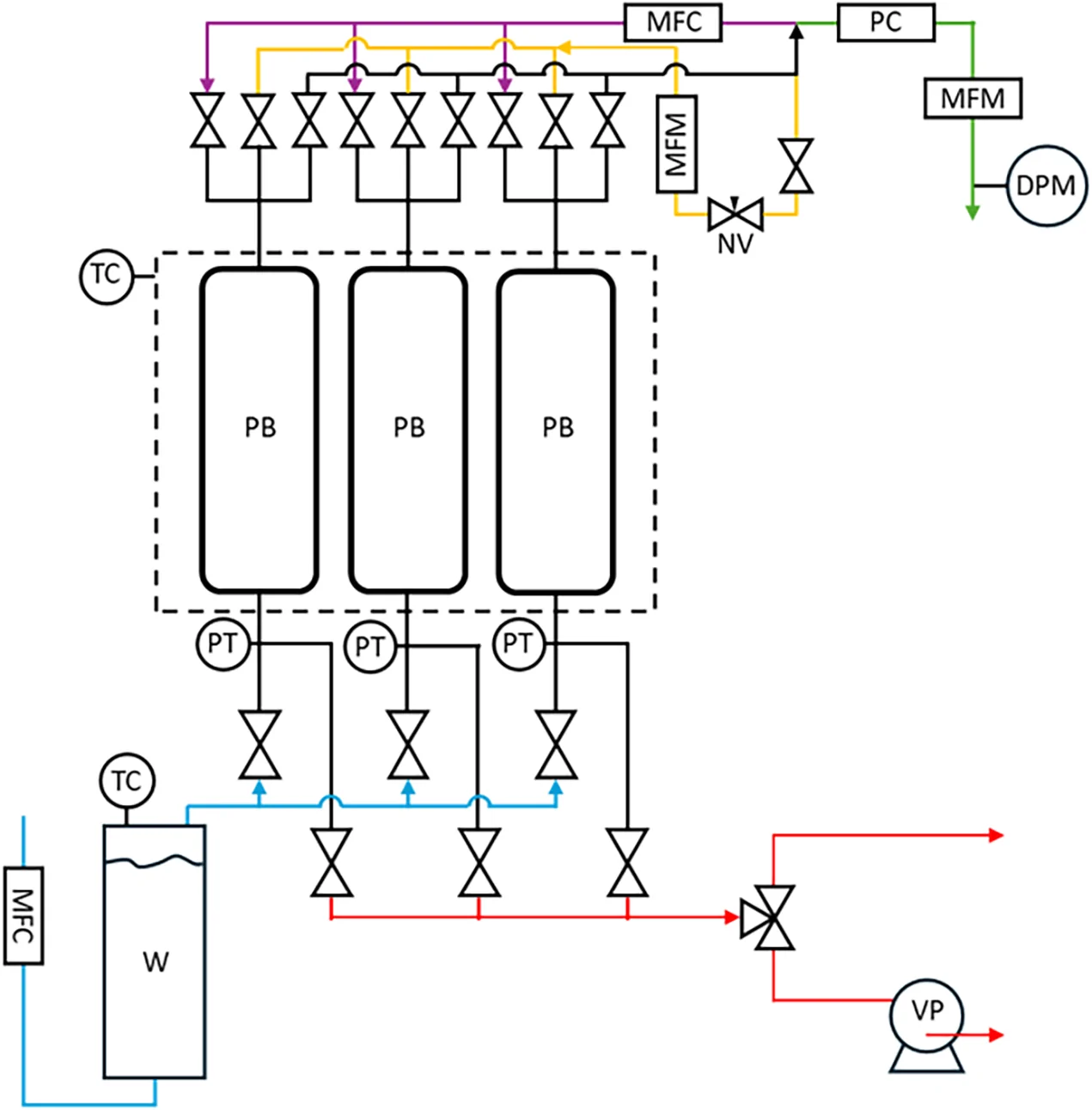
VPSA unit. DPM: dew point meter; MFC: mass flow controller; MFM: mass flow meter; NV: needle valve; PC: pressure controller; PB: packed bed; PT: pressure transducer; TC: thermocouple; VP: vacuum pump; W: water column.

### VPSA Screening Cycles

The VPSA cycles were structured into stages, which were segmented into steps—*cf*. Table 2 and Figure 3. The three packed beds of the VPSA unit are phase-shifted by 120° (one-third of the cycle period) to maintain steady, uninterrupted product delivery. Three cycles with different regeneration parameters were tested in screening experiments.

**Table 2:** Sequence of Elementary Steps of Screening Cycles

**Stage**	**I**			**II**			**III**		
	Cycle #1								
Step	1	2	3	4	5	6	7	8	9
Time/s	1	1	8998	1	1	8998	1	1	8998
PB 1	AD			DPE	BD	PG	PPE	BF	
PB 2	PPE	BF		AD			DPE	BD	PG
PB 3	DPE	BD	PG	PPE	BF		AD		

**Stage**	**I**			**II**			**III**		
Cycle #2									
Step	1	2	3	4	5	6	7	8	9
Time/s	1	1	8998	1	1	8998	1	1	8998
PB 1	AD			DPE	BD	EV	PPE	BF	
PB 2	PPE	BF		AD			DPE	BD	EV
PB 3	DPE	BD	EV	PPE	BF		AD		

**Stage**	**I**				**II**				**III**			
Cycle #3												
Step	1	2	3	4	5	6	7	8	9	10	11	12
Time/s	1	1	2	8996	1	1	2	8996	1	1	2	8996
PB 1	AD				DPE	BD	EV	PGV	PPE	BF		
PB 2	PPE	BF			AD				DPE	BD	EV	PGV
PB 3	DPE	BD	EV	PGV	PPE	BF			AD			

**Figure 3:**
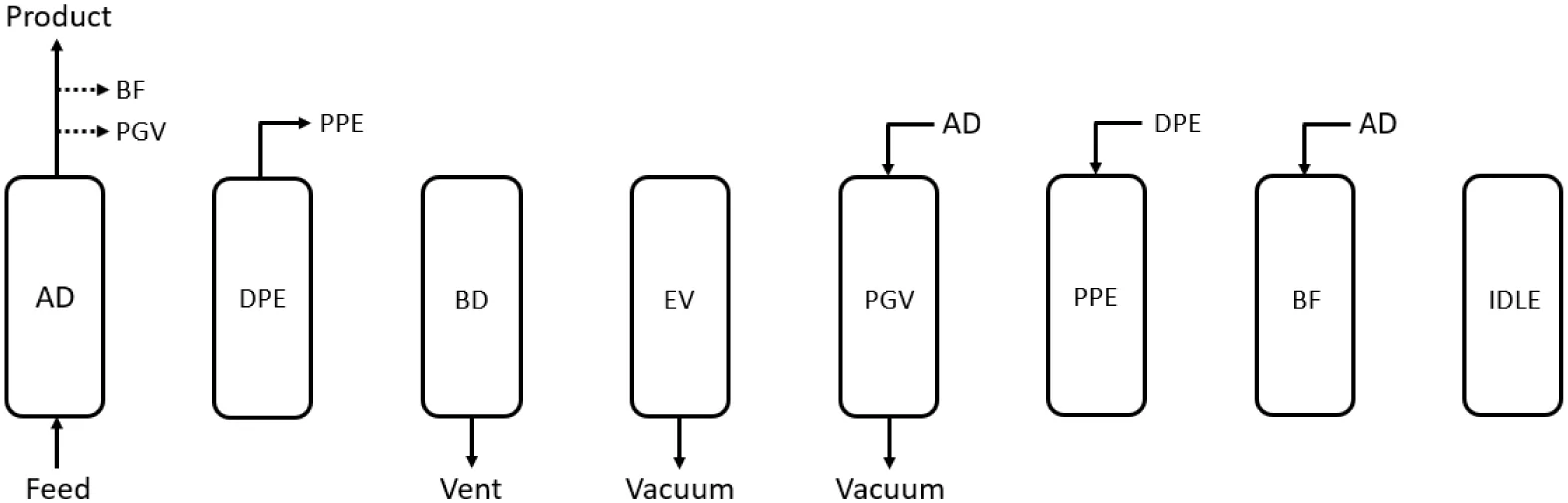
Representation of gas flow direction in all elementary steps.

Cycle #1—A representative sequence for packed bed 1 (PB 1) begins with Stage I, the production stage, during which the adsorption step (AD) is active, with the bed operating at the prescribed adsorption pressure to deliver the product. Stage II is the regeneration stage and consists of three steps in the respective sequence: first, depressurization by pressure equalization (DPE), coupled to the pressurization by pressure equalization (PPE) step of the PB 3, which transfers gas between beds to recover mechanical energy; second, blowdown (BD) to atmospheric pressure; and third, a countercurrent purge (PG) at the same pressure of BD, where *ca*. 1% of the product stream is used to regenerate the bed. Stage III restores PB 1 to adsorption conditions in two steps. PPE, now coupled to the DPE of PB 2 to reuse intermediate-pressure gas and reduce compression work, followed by a backfill (BF) step to reach the target adsorption pressure using product gas. The cumulative duration of all steps is chosen so that all three PBs execute the same AD → DPE-BD-PG → PPE-BF sequence with a constant 120° phase offset. Cycle #1 operates in PSA mode without regeneration aid from a vacuum pump and serves as a baseline for the next two cycles.

Cycle #2—This cycle is similar to Cycle #1 except that PG is replaced by an evacuation step (EV), without purge, to assess the efficacy of reduced pressure only for the removal of residual adsorbates.

Cycle #3—This cycle is similar to Cycles #1 and #2, but adds an extra step during Stage II, the purge under vacuum (PGV) step, where *ca*. 1% of the product stream is used to regenerate the bed under a reduced pressure. EV is shortened and serves the purpose of reducing pressure inside PB.

### VPSA Optimization Cycle and Evaluation

The optimization cycle implements cycle #3, using the countercurrent PGV step for packed bed regeneration; *cf*. Table 3. An additional step was added at the end of each stage; steps 5, 10, and 15 in Table 3. These are idle steps that are used whenever it is necessary to keep the duration of the three packed bed stages equal; this allows for closing the portion of the product stream used as backfill and purging from the producing PB 1 whose concentration front might have broken through. These steps prevent contact of water impurities from the purging gas in the regenerating PB 3 and from the backfill gas in the preparing PB 2. Such contact could result in a weaker regeneration and faster subsequent breakthroughs. The 120° phase offset is kept constant across packed beds, and its pressure history during a single optimization cycle in PB 1 is shown in Figure 4.

**Table 3:** Sequence of Elementary Steps of the VPSA Cycle

**Stage**	I					II					III				
**Step**	1	2	3	4	5	6	7	8	9	10	11	12	13	14	15
Time/s	1	1	2	*t*_opened_	*t*_closed_	1	1	2	*t*_opened_	*t*_closed_	1	1	2	*t*_opened_	*t*_closed_
**PB 1**	AD					DPE	BD	EV	PGV	EV	PPE	BF			IDLE
**PB 2**	PPE	BF			IDLE	AD					DPE	BD	EV	PGV	EV
**PB 3**	DPE	BD	EV	PGV	EV	PPE	BF			IDLE	AD				

**Figure 4:**
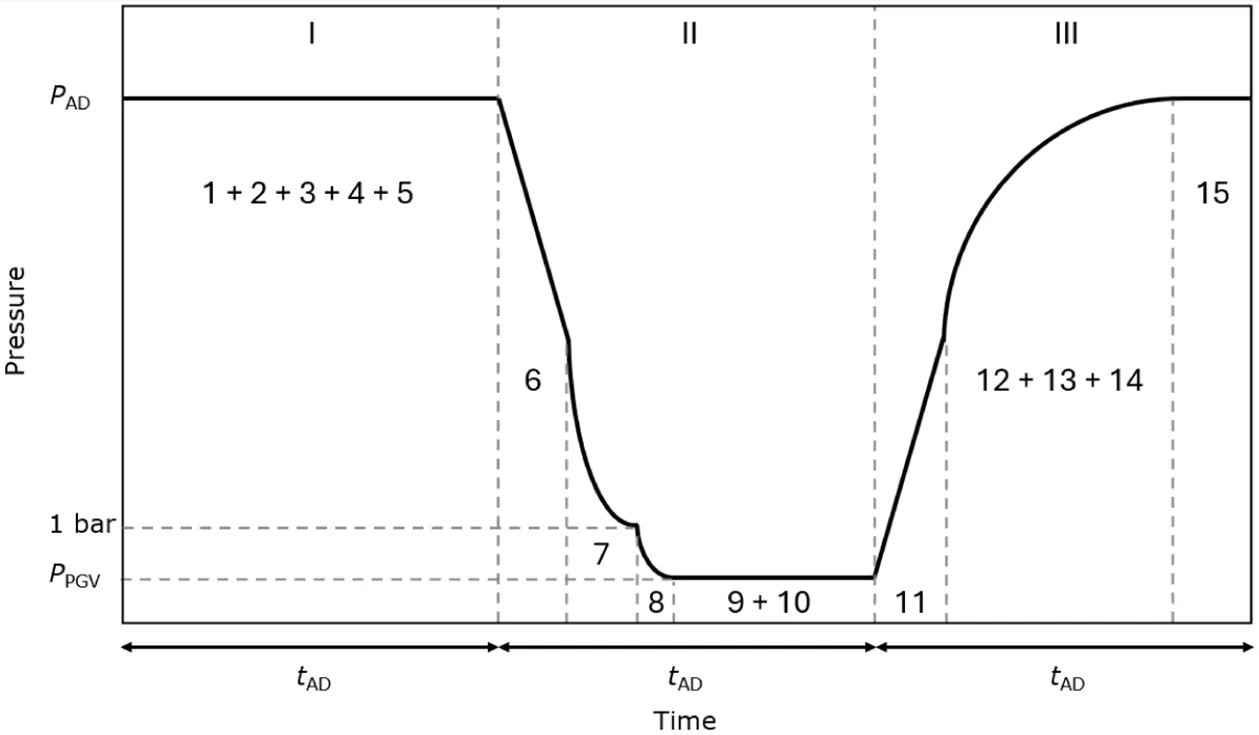
Pressure history of a packed bed in one cycle. Three major stages are represented: I) production stage, II) regeneration stage, and III) preparation stage.

The VPSA was assessed in terms of recovery ratio and productivity; the hydrogen dew point temperature was imposed as a boundary condition—the dried hydrogen average *T*_dp_ over an entire cycle. The productivity Ψ, was calculated in terms of hydrogen mass produced per adsorbent mass per cycle time according to eq 12. The recovery ratio *R*_rec_, is the ratio between product and feed hydrogen and it was computed according to eq 13.

12 $$\Psi =\frac{\rho_{H_{2}}F_{\mathrm{prod}}t_{\mathrm{cycle}}}{60\times m_{\mathrm{ads}}}$$

13 $$R_{\mathrm{rec}}=\frac{y_{H_{2},\mathrm{prod}}F_{\mathrm{prod}}}{y_{H_{2},\mathrm{feed}}F_{\mathrm{feed}}}$$

where $\rho_{H_{2}}$ is the density of H_2_ at normal conditions, *F*_prop_ is the volumetric flow rate of the product stream, *t*_cycle_ is time of one column cycle, and $y_{H_{2},\mathrm{prod}}$ is the volumetric fraction of H_2_ in the product stream.

### Design of Experiments

A design of experiments (DoE) based on response surface methodology (RSM) was conducted to identify an optimal operability region for the VPSA system. In RSM, the orthogonally structured set of experimental runs is used to fit an empirical quadratic model that relates input factors to response variables, thereby defining a continuous (one- or multidimensional) design space within which factor-response relationships can be interrogated.^42^ A Box-Behnken design (BBD), a circumscribed RSM variant that samples responses at factor combinations located on the midpoints of the experimental domain edges, was implemented. For a three-factor design, BBD evaluates conditions in which two variables are fixed at the extremes of their ranges while the third is held at its midpoint; replicate center points are included to quantify experimental error and detect potential drifts.^42^ The experimental results were used to estimate the coefficients of an empiric quadratic model presented in eq 14:

$$\begin{array}{l} ŷ=\beta_{0}+\beta_{1}\left(x_{1}-{\mathrm{x̅}}_{1}\right)+\beta_{2}\left(x_{2}-{\mathrm{x̅}}_{2}\right)+\beta_{3}\left(x_{3}-{\mathrm{x̅}}_{3}\right)\;+\\ \beta_{12}\left(x_{1}-{\mathrm{x̅}}_{1}\right)\left(x_{2}-{\mathrm{x̅}}_{2}\right)+\beta_{13}\left(x_{1}-{\mathrm{x̅}}_{1}\right)\left(x_{3}-{\mathrm{x̅}}_{3}\right)+\beta_{23}\left(x_{2}-{\mathrm{x̅}}_{2}\right)\left(x_{3}-{\mathrm{x̅}}_{3}\right)\;+\\ \beta_{11}{\left(x_{1}-{\mathrm{x̅}}_{1}\right)}^{2}+\beta_{22}{\left(x_{2}-{\mathrm{x̅}}_{2}\right)}^{2}+\beta_{33}{\left(x_{3}-{\mathrm{x̅}}_{3}\right)}^{2} \end{array}$$ 14

where *ŷ* is the predicted response, *β*_0_ is the intercept; *β*_1_, *β*_2_ and *β*_3_ are the linear (first-order) coefficients for factors *x*_1_, *x*_2_ and *x*_3_, *β*_12_, *β*_13_ and *β*_23_ are the two-factor interaction coefficients quantifying cross-effects; *β*_11_, *β*_22_ and *β*_33_ are the quadratic (second-order) coefficients capturing curvature.

The investigated operating variables were the adsorption pressure, *P*_AD_, the adsorption step time, *t*_AD_, and the time fraction, *θ*_closed_, corresponding to the fraction of *t*_AD_ during which the product stream is isolated from the equalization and purge steps and withdrawn exclusively as product. This variable was introduced to account for the possible decrease in product purity toward the end of the adsorption step. To avoid contamination of the remaining columns with lower-purity hydrogen, the backfill and purge streams were terminated prior to the end of *t*_AD_, according to the selected value of *θ*_closed_:

15 $$\theta_{\mathrm{closed}}=t_{\mathrm{closed}}/t_{\mathrm{AD}}$$

where *t*_closed_ is the duration of steps 5, 10, and 15. Since *θ*_closed_ and *t*_AD_ are input variables, *t*_opened_ takes the remaining duration from eq 16.

16 $$t_{\mathrm{opened}}=t_{\mathrm{AD}}-\left(t_{\mathrm{DPE}}+t_{\mathrm{BD}}+t_{\mathrm{EV}}+t_{\mathrm{PGV}}+t_{\mathrm{EV}}\right)=t_{\mathrm{AD}}-\left(t_{\mathrm{PPE}}+t_{\mathrm{BF}}+t_{\mathrm{IDLE}}\right)=t_{\mathrm{AD}}-\left(4+t_{\mathrm{closed}}\right)$$

The chosen factors were investigated applying a three-level, equidistant design in which the low, center and high settings are coded as “–”, “0” and “+”, respectively. The corresponding actual (uncoded) values for each factor are listed in Table 4.

**Table 4:** Range Values for the Input Variables in DoE

	–	0	+
***x***_**1**_:*t*_**AD**_ **/h**	1.5	2.5	3.5
***x***_**2**_:*P*_**AD**_ **/bar**	4	6	8
***x***_**3**_ **:θ**_**closed**_	0	0.0714	0.1428

The factor ranges were selected based on preliminary screening experiments. Optimization within the chosen ranges is governed by two criteria. First, the purity of the product stream is enforced via a dewpoint specification of *T*_dp_ = −67.5 °C with a tolerance of ±2 °C, to ensure *T*_dp_ < −65.5 °C (the contamination limit). Second, subject to this purity constraint, the objective is to maximize feed recovery. The common operating conditions to all experiments and the packed-bed characteristics of the VPSA unit are summarized in Table S2.

The experimental factors were evaluated for their statistical significance in explaining the response variables by hypothesis tests on the regression coefficients (*H*_0_ : *β* = 0) . Terms with *p* < 0.05 were deemed significant—equivalently, a 95% confidence criterion was applied for retention in the model. After computing the RSM optimum, a sensitivity analysis was performed on the regeneration pressure while holding all other factors at their optimal values, to quantify its effect on *T*_dp_ and *R*_rec_. The regeneration pressure (subatmospheric pressure) was varied through 0.208, 0.099, 0.054, and 0.025 bar during the sensitivity analysis phase to the optimal experiment to measure its impact on *T*_dp_ and *R*_rec_.

### Economic Analysis

Although experimental testing is essential to assess the feasibility of a process within a research context, a process that also has commercial feasibility needs to be cost-competitive. A theoretical appraisal of a 300 kg_H_2__·h^–1^ system, estimating capital expenditure (CAPEX) and operational expenditure (OPEX) under defined assumptions, was therefore performed. The lower heating value of hydrogen was taken as 120 MJ·kg^–1^, corresponding to a 10 MW dryer. It was further assumed that the cycle-level transport and adsorption phenomena remain invariant with scale; hence, the unit volume was scaled in proportion to the feed flow rate, and the key performance indicators *T*_dp_ and *R*_rec_ were taken to be unchanged from the laboratory system. These assumptions are discussed in more detail later in the text. Since the equations used for the calculation of expenditures and the levelized cost of hydrogen drying (LCOHD) are not part of the core scientific discussion, they are described in the Supporting Information. Table S3 summarizes the relevant parameters for this subsection.^43,44^

## Results and Discussion

### Isotherm Characterization

Figure 5 plots the adsorption isotherms of water vapor and hydrogen on activated alumina at three temperatures: 20 °C, 30 °C, and 40 °C.

**Figure 5:**
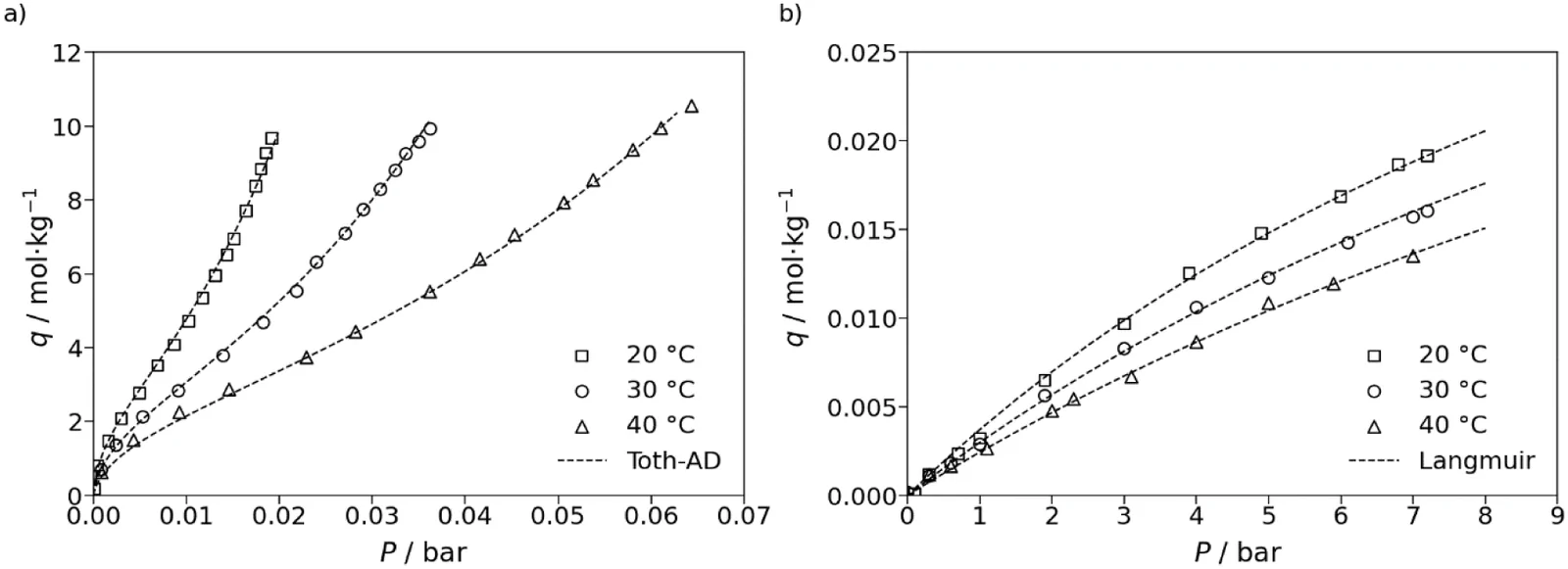
Adsorption isotherms on activated alumina at 20 °C, 30 °C, and 40 °C for: a) water vapor; b) hydrogen. Dashed lines are the Toth-AD fitting equation for water vapor and the Langmuir fitting equation for hydrogen.

The water adsorption isotherm is Type IV according to the International Union of Pure and Applied Chemistry classification, characteristic of mesoporous adsorbents (multilayer adsorption followed by capillary condensation). This behavior, including a hysteresis loop, is typical of activated alumina.^45,46^

Water adsorption on activated alumina typically proceeds in four stages. First, chemisorption occurs as water dissociates to neutralize acidic (Lewis-acid Al) and basic (O^2–^) sites created by unbalanced aluminum–oxygen bonds.^46^ Second, strongly hydrogen-bonded layers form with restricted, “ice-like” mobility. The third stage concerns the onset of pore-filling/capillary condensation in mesopores (the transitional regime). Finally, weakly bound multilayers develop with liquid-like mobility.^45,47,48^

In contrast, the hydrogen isotherm shows a low adsorption capacity across all measured temperatures.

Both models were fitted to the experimental data by minimizing the root-mean-square error (RMSE), and the resulting parameters are reported in Table 5 (Langmuir) and Table 6 (Toth-AD). Fitting the Langmuir model was straightforward, given its small number of parameters—two parameters plus one to account for the temperature (eqs 1 and 3). Although the Langmuir equation provides a good fit to the experimental data, this should not be interpreted as evidence that the Langmuir adsorption model is physically valid for the system under study. Consequently, conclusions should not be drawn directly from the fitted parameters, particularly *q*_m_, which represents the monolayer adsorption capacity only if the assumptions of the Langmuir model are satisfied. In the present case, the limited pressure range investigated does not allow validation of these assumptions and therefore does not support a physical interpretation of *q*_m_. Rather, *q*_m_ should be regarded solely as an empirical fitting parameter.

**Table 5:** Langmuir Model Parameters for Hydrogen Adsorption in Activated Alumina

*q*_m_/mol·kg^–1^	$b_{1}^{0}$ /bar^–1^	*Q*_1_/kJ·mol^–1^	RMSE/mol·kg^–1^
0.057	0.1178	17.26	0.0003

**Table 6:** Toth Aranovich-Donohue Model Parameters for Water Vapor Adsorption in Activated Alumina

*q*_m_/mol·kg^–1^	*f* ^0^	*α*	$b_{2}^{0}$ /bar^–1^	*Q*_2_/kJ·mol^–1^	$b_{3}^{0}$ /bar^–1^	*Q*_3_/kJ·mol^–1^	*n*	RMSE/mol·kg^–1^
13.5	0.286	0.0073	3262.0	46.17	7.1	41.37	23.6	0.21

By contrast, the Toth-AD model required estimating six parameters plus two to account for the temperature (eqs 2, 3 and 4), increasing the risk of overfitting and potentially yielding parameters with limited physical meaning.

To assess the physical plausibility of the Toth-AD fit, the isosteric heat of adsorption was computed independently using eq 6. The model is deemed physically consistent when the heats on the Toth side (*Q*_2_) and on the AD side (*Q*_3_) exceeded the heat of vaporization (*Q*_vap_ ≈ 42 kJ·mol^–1^) and *Q*_2_ > *Q*_3_. These criteria reflect the expected layering behavior: initial adsorption layers, being closer to the surface, should experience stronger adsorbent–adsorbate interactions (higher adsorption heat), whereas the final layer should approach reversibility, where interactions are predominantly between water molecules, with the heat of adsorption converging to the heat of vaporization. Figure 6 illustrates how the heat of adsorption evolves with adsorption capacity.

**Figure 6:**
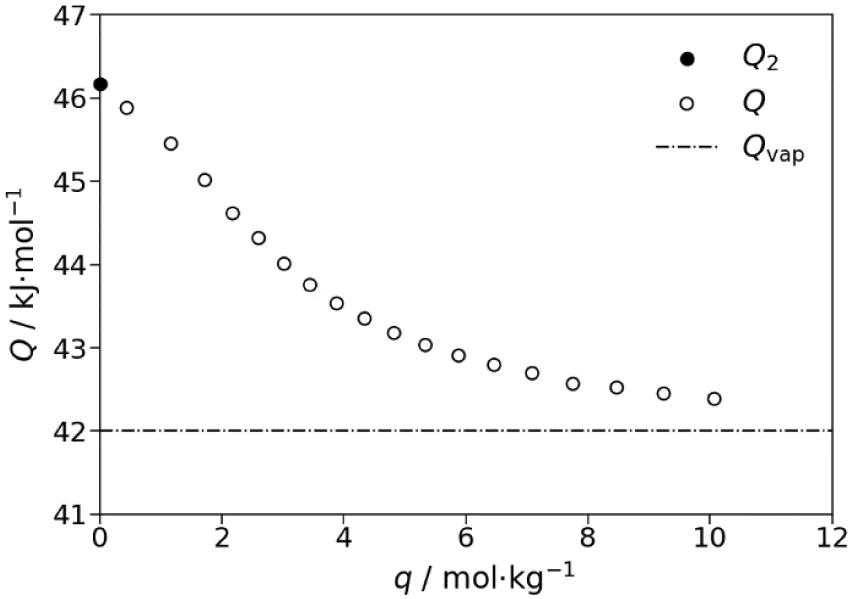
Experimental heat of adsorption of water vapor on the activated alumina as a function of loading obtained from the integrated form of Clausius–Clapeyron.

The Toth-AD equation satisfies the criteria for a thermodynamically valid description of water adsorption. It reduces to Henry’s law at low concentrations and captures the expected decrease in adsorption energy as additional layers are adsorbed.

The experimental results do not indicate the existence of chemisorption. To confirm that, the sample was regenerated under vacuum (25 mbar overnight) at 20 °C, and an additional isotherm was obtained at the same temperature. The new isotherm matched the previous one, which evidence the absence of chemisorption. This test was repeated twice with the same result.

The absence of chemisorption was assigned to a low value of *Q*_2_: 46.2 kJ·mol^–1^, insufficient for the occurrence of chemisorption.^30^ The comparatively modest *Q*_2_ may also account for the lower water uptake relative to other activated alumina reported in the literature.^37,49^ Finally, as noted by Serbezov,^50^ the three water isotherms collapse onto a single curve when plotted against the relative humidity, as illustrated in Figure 7.

**Figure 7:**
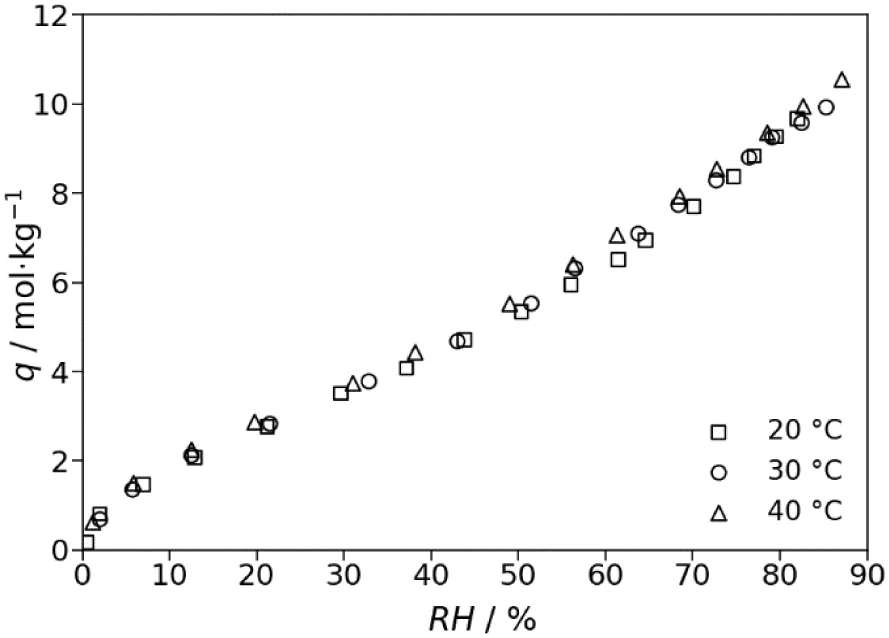
Experimental adsorption isotherms of water vapor as a function of relative humidity for 20 °C, 30 °C and 40 °C.

### Breakthrough Characterization

Hydrogen feed streams, with different relative humidities, pressures, and temperatures, and for different regeneration times of the bed, were tested to study the dynamics of adsorption. Breakthrough time was defined as the instant where the *T*_dp_ exceeded −65.5 °C. The experimental results are presented in Table S4.

### Concentration Fronts

Figure 8 compares breakthroughs BT1, BT2, and BT3. All these experiments were conducted at the same temperature (28.5 °C), for different RH (50%, 70% and 80%), and consequently *T*_dp_. Before each test, the adsorption bed was regenerated overnight at 28.5 °C under reduced pressure (*ca*. 25 mbar) and a 0.015 L·min^–1^ purging flow of dry hydrogen.

**Figure 8:**
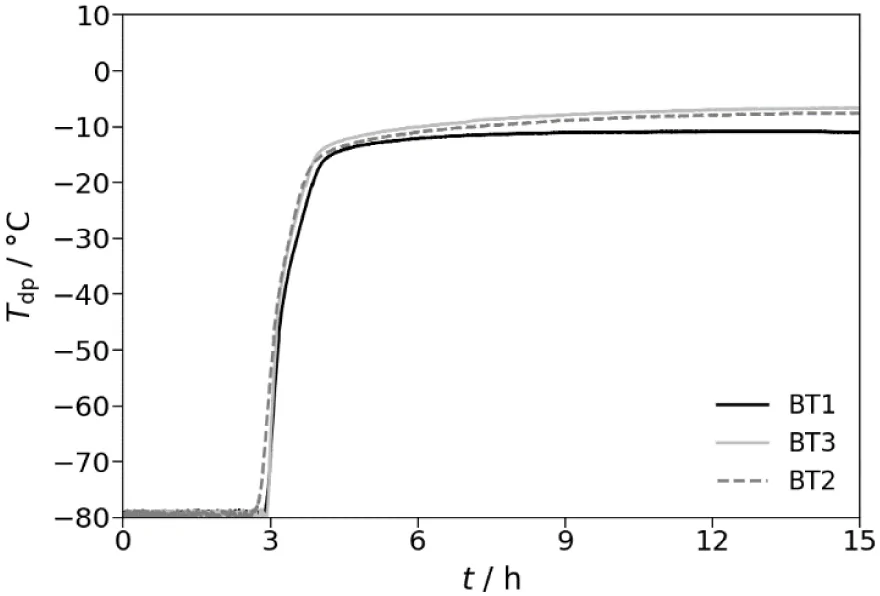
Breakthrough curves for the experiments BT1, BT2 and BT3.

As RH increases, *q*_s_ also increases, matching the values predicted by the Toth-AD equation. However, *η*_ads_ decreases from 87.6% to 79.2%. This drop was attributed to the increasingly dispersive nature of water adsorption as relative humidity increases from BT1 to BT3, hence the concentration front suffers greater axial dispersion.^51^

Park and Knaebel^52^ examined the influence of RH on water-vapor breakthrough in silica gels and observed that, at high RH, discontinuities emerge in the concentration front due to compressive-to-dispersive transitions in the equilibrium isotherm.

A similar behavior was observed here. The equilibrium isotherm exhibits an inflection point between 20% and 30% RH. Figure 9 illustrates this using the Toth-AD model at 28.5 °C and its first derivative. The transition from a sharp shock wave to a dispersive front occurs near −17.5 °C *T*_dp_ (26.9% RH under the experimental conditions). Beyond this point, the concentration front develops a lagging tail that can take hours to fully develop and becomes progressively steeper with increasing RH.

**Figure 9:**
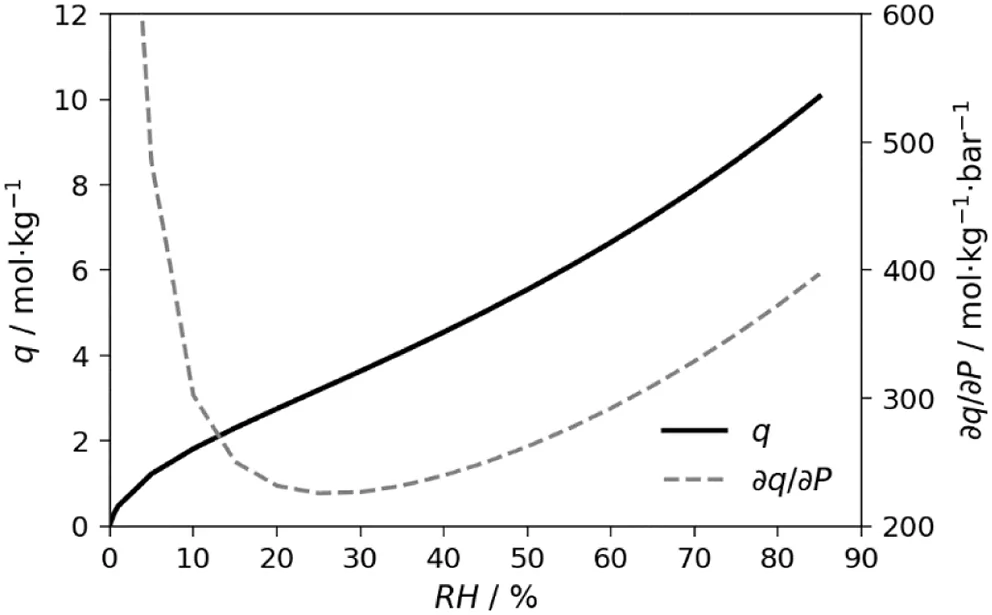
Toth-AD model for water vapor adsorption equilibrium at 28.5 °C versus its first derivative against equilibrium pressure.

Figure 10 plots experiments BT4 and BT5, both conducted at 4 bar. BT4 was performed at 70% RH, whereas BT5 was conducted at 35% RH. In both cases, *q*_s_ agrees with the values predicted by the equilibrium adsorption isotherms (*cf*. Table S4). However, *η*_ads_ was markedly lower in BT4 (72.5%) than in BT5 (79.6%). This difference is consistent with the compressive-dispersive behavior of the adsorption isotherm because 35% RH (BT5) lies near the isotherm’s inflection point, hence the lagging tail of the concentration front is substantially diminished. Despite BT4 and BT2 being run at 70% RH, BT2 exhibited higher efficiency (*η*_ads_ = 81.7%). This indicates that total pressure, and thus the superficial velocity within the packed bed, strongly affects the separation performance.

**Figure 10:**
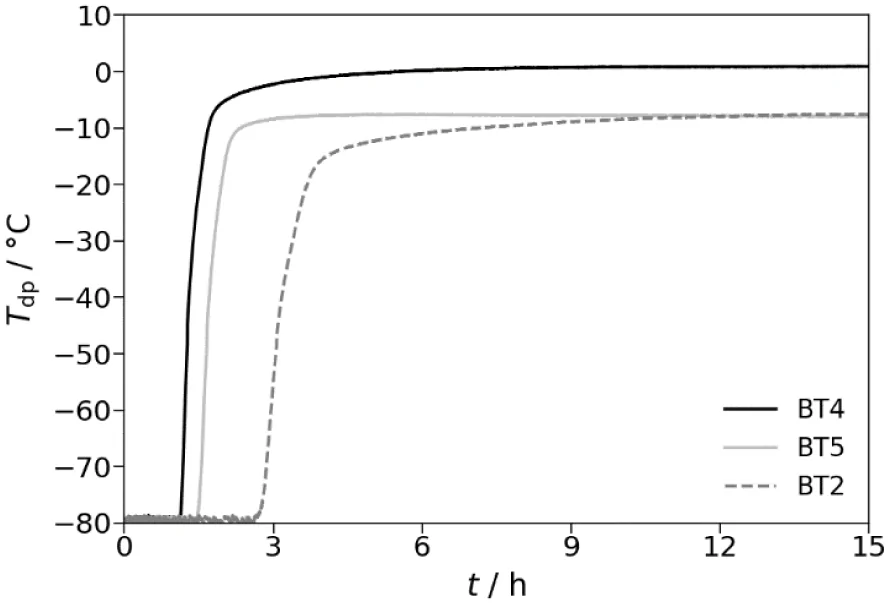
Breakthrough curves for the experiments BT4 and BT5. BT2 included for comparison.

Figure 11 plots the results of experiments BT6 and BT7, which differ from the other experiments in terms of regeneration time; in experiments BT6 and BT7 the bed was regenerated for only 1.5 and 2.5 h, respectively, after complete saturation at 28.5 °C. These shorter regeneration times enabled to probe how incomplete regeneration shapes the concentration history during adsorption breakthroughs. In experiment BT6, a noteworthy behavior emerged: at the outlet, *T*_dp_ continued to decline, indicating that the entering fraction of the bed was regenerated but not the latter fraction; the feeding hydrogen was drying at the entering fraction of the column and, as it was entering the latter fraction, it was purging it. As adsorption proceeded and the adsorbent approached saturation, this drying effect diminished and *T*_dp_ subsequently began to rise.

**Figure 11:**
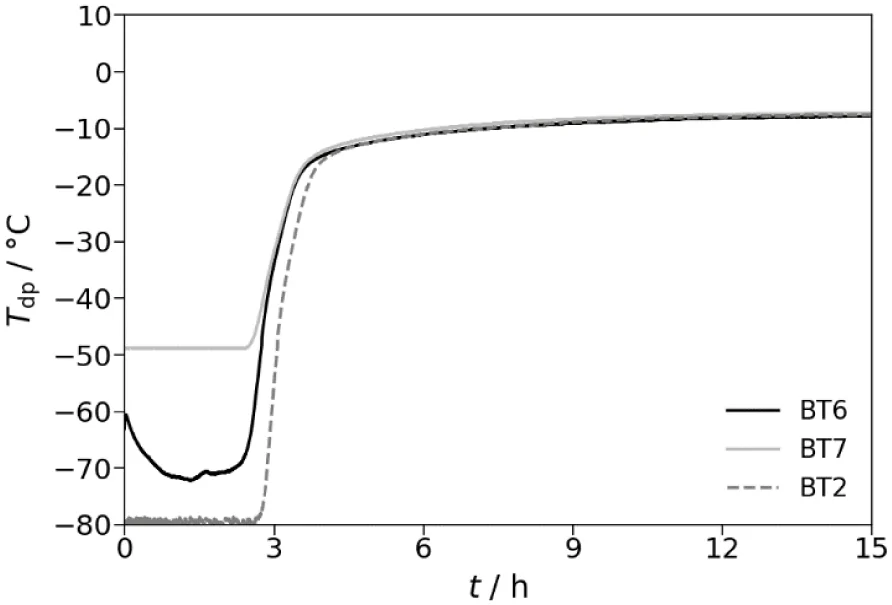
Breakthrough experiments BT6 and BT7. BT2 included for comparison.

Lastly, Figure 12compares experiments BT8 and BT2, performed at different temperatures, with the remaining operating variables held constant. Given the same RH, the water vapor capacity is expectedly equal for both experiments (*cf*. Figure 7); however, the absolute humidity at 23 °C (0.8 mol·m_N_^–3^), is lower than at 28.5 °C (1.1 mol·m_N_^–3^). Consequently, less water was fed to the bed at the lower temperature, delaying saturation and extending the breakthrough time.

**Figure 12:**
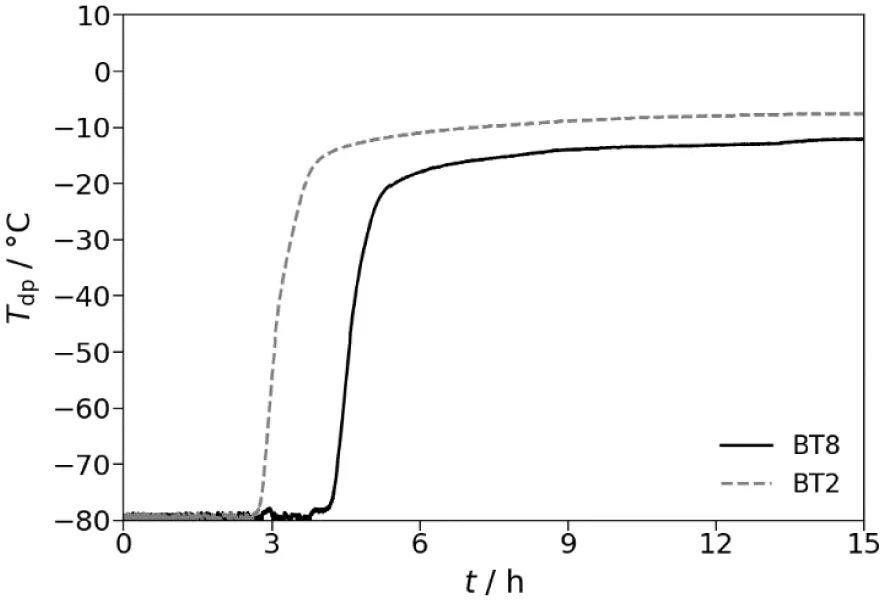
Breakthrough curve for the experiment BT8. BT2 included for comparison.

### Temperature Fronts

The temperature was monitored during all experiments at relative axial positions of 0.1, 0.5, and 0.9 along the adsorbent bed. Figure 13 shows the temperature histories for experiments BT1, BT2, and BT3, in which only the relative humidity was varied. Increasing the relative humidity from 50% to 70% and 80% led to progressively higher temperature peaks, with maximum temperature rises of approximately 2.5 °C, 3.5 °C, and 4 °C for BT1, BT2, and BT3, respectively. This trend reflects the exothermic nature of adsorption and the larger amount of water adsorbed at higher humidity levels. Although broader adsorption fronts and greater heat release were observed at higher relative humidity, no significant spreading of the thermal front occurred, as the temperature peaks appeared at similar times in all cases.

**Figure 13:**
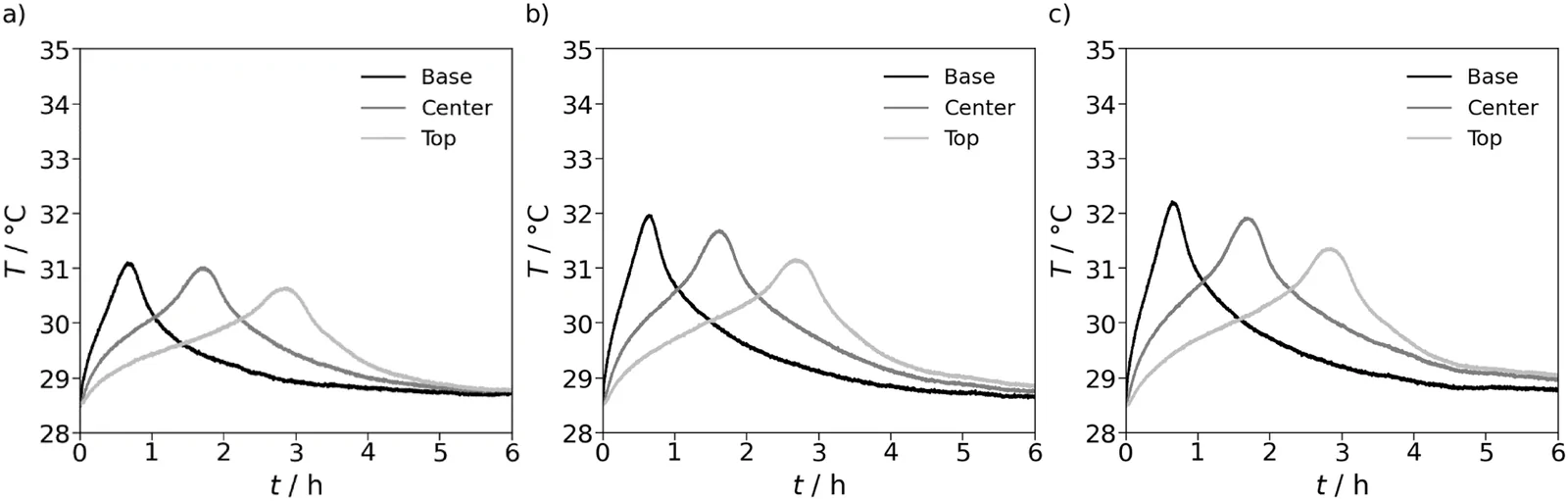
Adsorption temperature histories for experiments: a) BT1; b) BT2; c) BT3.

The desorption temperature histories for experiments BT1 to BT3 exhibit increasingly pronounced temperature variations as the loaded humidity increases, under operating conditions of 25 mbar and 15 mL·min^–1^. Moreover, the temperature remains lowered for longer periods. Figure 14 presents the corresponding temperature histories during desorption.

**Figure 14:**
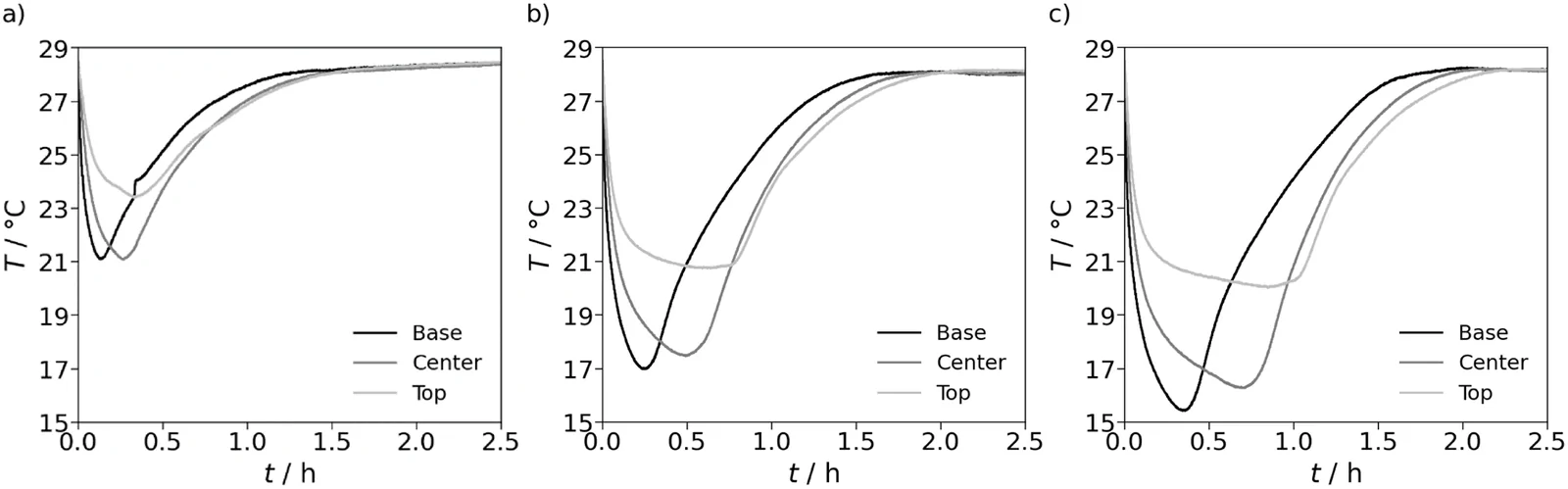
Desorption temperature histories for experiments: a) BT1; b) BT2; c) BT3.

The comparison of experiments BT2, BT4, and BT5, presented in Figure 15, provides further insights. The effect of lower feeding pressure (BT4 vs. BT2) yielded a less efficient heat dissipation, leading to an increase in internal temperature of approximately 6.5 °C. Since the operating pressure in BT4 is half that of BT2, while maintaining the same molar flow rate, the superficial velocity within the adsorbent bed in BT4 is two times faster. This requires rapid intraparticle diffusion of water vapor to maintain a sharp concentration front. Experiment BT5 was conducted to evaluate the effect of absolute humidity. Although BT2 and BT5 have the same dew point at 1 bar, different pressure conditions yield distinct relative humidities (70% vs. 35% RH). Consequently, similar temperature levels are observed in both experiments, with a slight increase in BT5 at midbed (relative position 0.5), which may be attributed to a higher average adsorption energy at 35% RH in BT5 (*cf*. Figure 6). The adsorption efficiency at BT5 conditions remains high (79.6%), consistent with the less dispersive adsorption front associated with its relative humidity conditions. Furthermore, the propagation speed of the temperature front appears to scale with pressure, as the temperature peaks in BT4 and BT5 develop approximately half as fast as in BT2.

**Figure 15:**
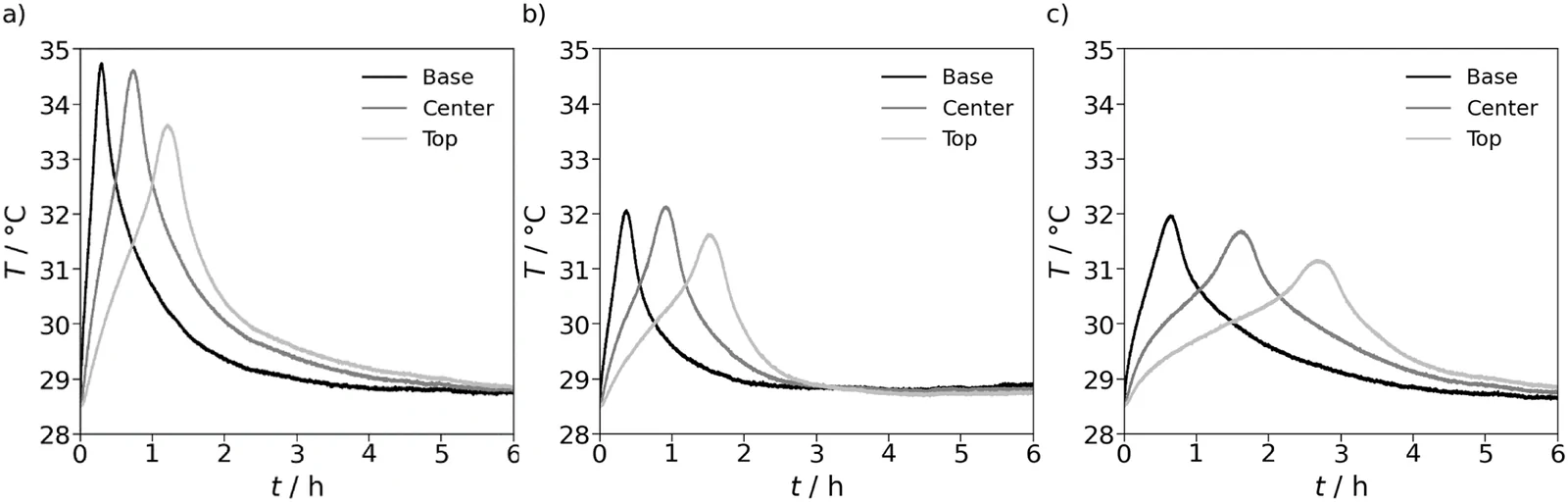
Adsorption temperature histories for experiments: a) BT4; b) BT5; c) BT2 (for comparison).

The temperature history of BT8, shown in Figure 16, exhibits a lower heat release (*ca*. 2.5 °C) than BT2 (*ca*. 3.5 °C), indicating a lower adsorption rate associated with reduced absolute humidity.

**Figure 16:**
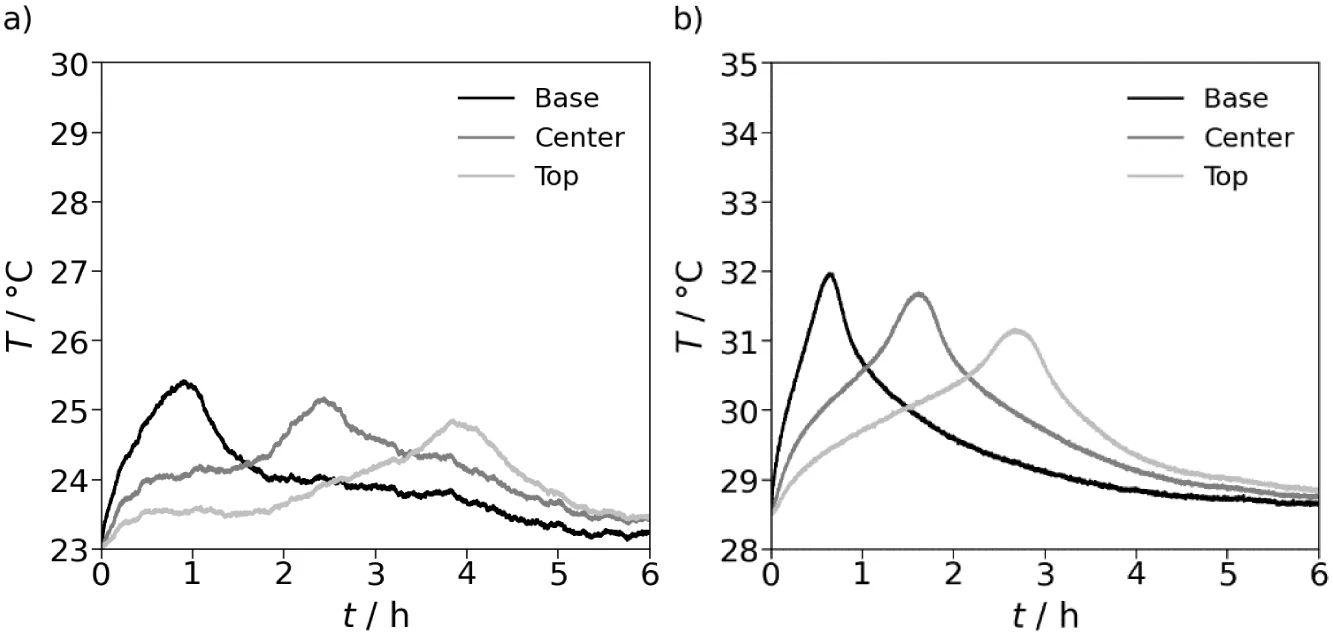
Adsorption temperature histories for experiments: a) BT8; b) BT2 (for comparison).

### VPSA Screening Experiments

Two main findings emerge from the VPSA screening tests in Table 7. First, the regeneration strategy strongly governs the purity of the product stream. Neither purging nor pressure reduction alone reaches a *T*_dp_ < −65.5 °C. When combined, however, *T*_dp_ dropped to −70.05 °C, indicating a synergistic effect. Vacuum regeneration reduces the total pressure and the partial pressure, making it more effective than purging alone. Adding a purge to the vacuum further accelerates desorption by lowering the humidity partial pressure and providing convective drag for dried hydrogen through the bed. Second, high hydrogen recovery was achieved: *R*_rec_ > 98%. The highest recovery was observed with cycle #2 (evacuation without purge), where hydrogen losses are limited to the holdup remaining after the equalization step. By contrast, cycle #3 (purge under vacuum) showed the lowest recovery because additional hydrogen is vented during the EV step to reduce pressure below atmospheric comparatively to cycle #1. These findings set the choice of the regeneration method for the optimization experiments.

**Table 7:** Operating Conditions and Results for the Cycles Used in the Screening Tests—P/F Is the Ratio of the Purge and Feed Volume

Cycle	*t*_AD_/h	*F*_feed_/L_N_·min^–1^	P/F	*P*_AD_/bar	*P*_regen_/bar	*R*_rec_ (%)	*T*_dp_/°C
**PG**	2.5	1.5	1%	8	1	98.61	–9.62
**EV**	2.5	1.5	0%	8	0.025	99.37	–49.01
**PGV**	2.5	1.5	1%	8	0.025	98.34	–70.05

### VPSA Optimization and Design of Experiments

In day-to-day operation, a unit designed to deliver a dew point of −65.5 °C may perform slightly worse; for example, if the ambient temperature is higher than assumed. Therefore, the unit should be specified for a slightly lower (more stringent) dew point to ensure robustness. In the DoE, the established desirability function imposes a *T*_dp_ between −65.5 °C and −67.5 °C, for the maximum *R*_rec_; All runs from the DoE were conducted at a regeneration pressure of 0.025 bar with a 1% P/F. As expected, hydrogen recovery remained high (>98%), where tighter process control compared to the screening experiments reduced losses; in the experiment performed using the determined optimum operating conditions (opt), only 0.02% was lost to venting and uncontrolled leaks. The DoE operating conditions and the resulting dew point temperatures and recoveries are presented in Table S6.

In the quadratic empirical model fitted to the data, the first-order *P*_AD_ factor (coefficient *β*_2_ 
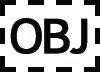
) is significant (p-value < 0.05), consistent with the breakthrough experiments: greater pressures increase packed bed efficiency and extend the time to concentration front break. The first-order *t*_AD_ term (*β*_1_) and the quadratic $t_{\mathrm{AD}}^{2}$ term (*β*_11_) are also significant since adsorption times can be shortened to prevent breakthrough.

The factor *θ*_closed_ $\left(\theta_{\mathrm{closed}}=\frac{t_{\mathrm{closed}}}{t_{\mathrm{AD}}}\right)$ was not statistically significant, and thus it was removed from the interpolating polynomial. Within the searched factors’ domain, *θ*_closed_ had a negligible effect on product purity, indicating that the breakthrough contamination had little impact. The low pressure prevailing in the regenerating PB 2 likely provides sufficient protection against contamination from PB 1 at the end of the adsorption step, since PB 2 is already mostly regenerated. Conversely, when PB 3 nears operating pressure toward the end of its preparation stage, water diffusion through the backfill line is likely the dominant contamination mechanism.

The coefficients for the quadratic factors of the RSM model are shown in Table 8. Applying the coefficients in Table 8 into eq 13 transforms it into eqs 17 for *T̂*_dp_ and 18 for *R̂*_rec_.

17 $${\mathrm{T̂}}_{\mathrm{dp}}=-55.508+14.447\times \left(t_{\mathrm{AD}}-2.5\right)-10.024\times \left(P_{\mathrm{AD}}-6\right)-1.309\times {\left(t_{\mathrm{AD}}-2.5\right)}^{2}$$

18 $${\mathrm{R̂}}_{\mathrm{rec}}=98.983+3.056\times 10^{-3}\times \left(t_{\mathrm{AD}}-2.5\right)-1.852\times 10^{-3}\times \left(P_{\mathrm{AD}}-6\right)-2.783\times 10^{-3}{\left(t_{\mathrm{AD}}-2.5\right)}^{2}$$

**Table 8:** Values and Statistical Validity of the Quadratic BBD Model Coefficients

Coefficient	*T̂*_dp_	p-value	*R̂*_rec_	p-value
**β**_**0**_	–55.508	<0.0001	98.983	<0.0001
**β**_**1**_	14.447	<0.0001	3.056 × 10^–3^	0.0012
**β**_**2**_	–10.024	<0.0001	–1.852 × 10^–3^	0.0001
**β**_**11**_	–1.309	0.0446	–2.783 × 10^–3^	0.0177

For the optimum operating conditions *t*_AD_ = 2.95 h and *P*_AD_ = 7.8 bar—*cf*. Table 9, the model predicts −68.28 < *T*_dp_ °C < −66.36 and a 98.9790 < *R̂*_rec_% < 98.9824. The corresponding experimental values were *T*_dp_ = −66.8 °C and *R*_rec_ = 98.98%, both within the error limits. The productivity obtained for the optimum was 3.92 kg_H_2__·kg_ads_^–1^. From eq 12, higher productivities are mostly dependent on a longer *t*_cycle_, since *F*_feed_ is constant and $\rho_{H_{2}}$ is mostly constant at the tested conditions. Hence, a longer optimal *t*_AD_ will yield a more productive VPSA.

**Table 9:** Operating Parameters and Results for the Estimated and Experimental Optimal Point

	*t*_AD_/h	*P*_AD_/bar	*R*_rec_ (%)	*T*_dp_/°C	*Ψ*/kgH_2_·kg_ads_^–1^
**Model**	2.95	7.8	98.9807 ± 0.0017	–67.32 ± 1.06	-
**Experimental**			98.9803	–66.76	3.92

The response variables *T*_dp_ and *R*_rec_ are plotted in Figure 17, where the quadratic nature of *R*_rec_ is more pronounced than *T*_dp_ as described by a lower absolute *β*_11_ that is statistically close to the cutoff value (p-value > 0.05).

**Figure 17:**
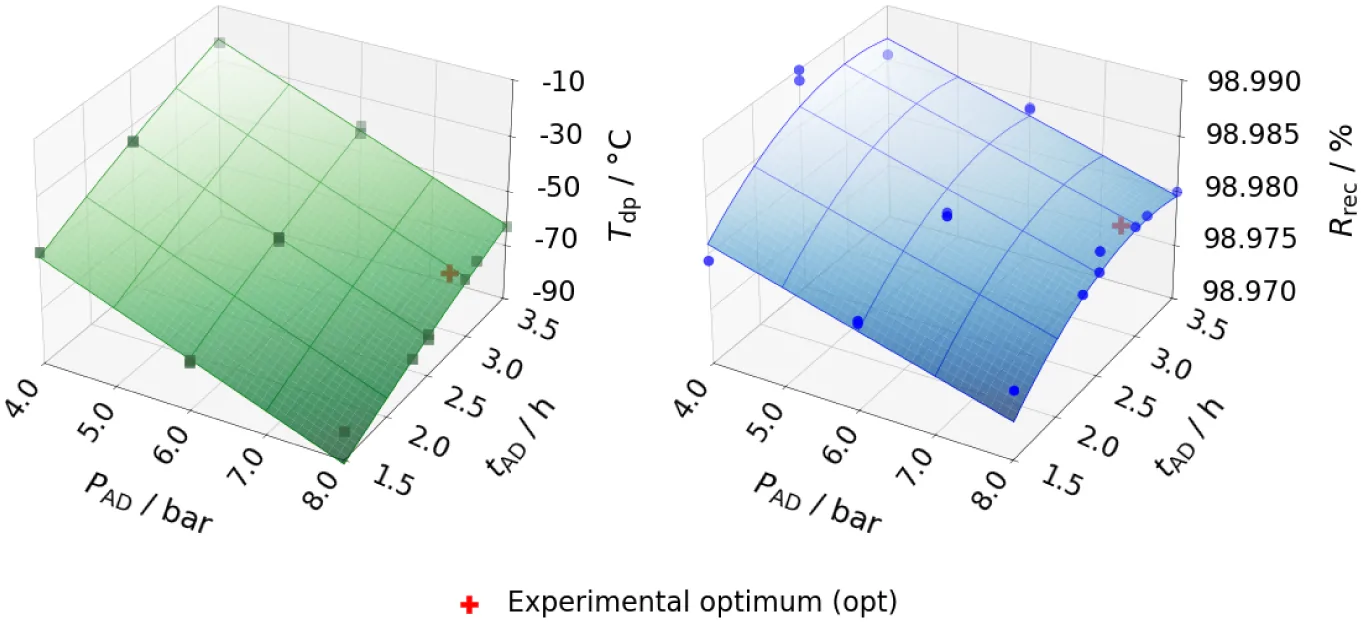
Surface plots of the response variables, experimental BBD points, and opt experiment.

The Studentized residuals of *T*_dp_ and *R*_rec_ are plotted in Figure 18. All experiments are within the individual and Bonferroni outlier limits, and no apparent bias is visible in both responses.

**Figure 18:**
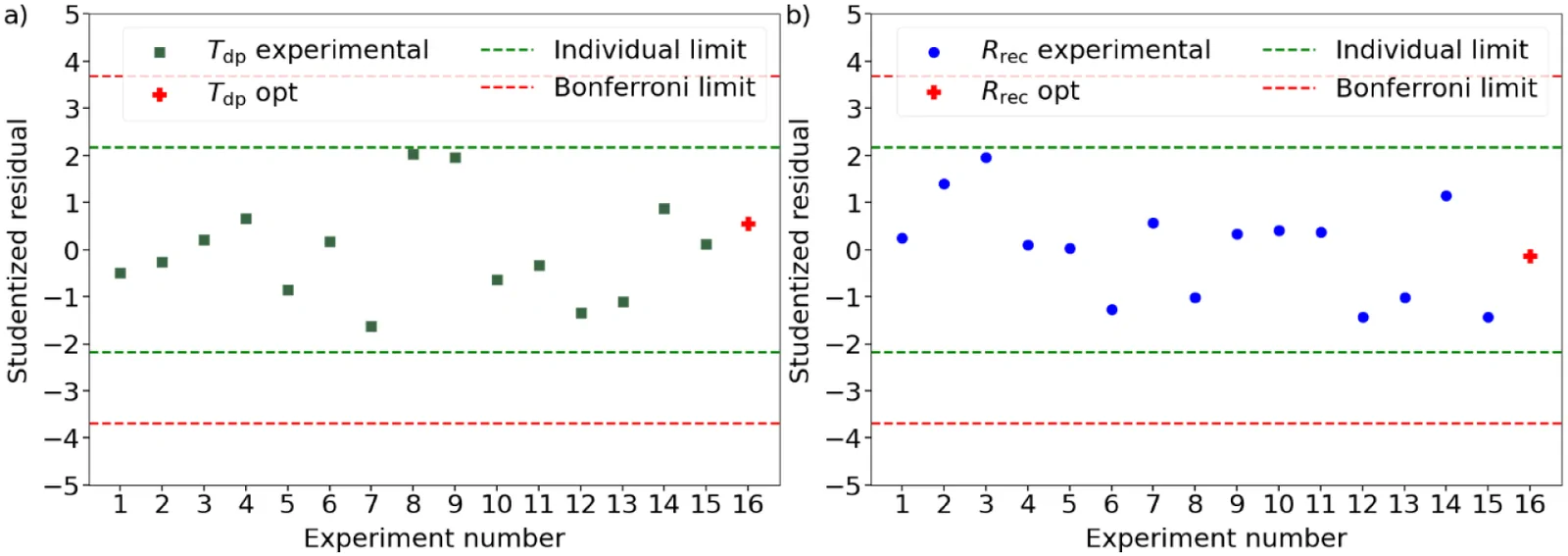
Studentized residuals of response variables for the experimental BBD points and opt experiment for: a) T_dp_ and b) R_rec_.

### Effect of Regeneration Pressure

Understanding how vacuum pressure during the regeneration step affects the performance of the VPSA is crucial for selecting a suitable vacuum pump. Lower pressures require more powerful and more expensive vacuum pumps. The results are shown in Figure 19 from a pressure range between 25 mbar and 208 mbar. As *P*_PGV_ decreases, *T*_dp_ also decreases in an accelerated way. The vacuum pressure should then be set to the highest value (lowest cost) that meets the required dew point temperature < −65.5 °C and, preferably, <−67.5 °C, with a recovery > 98%, which in this case corresponds to the lowest pressure: 25 mbar.

**Figure 19:**
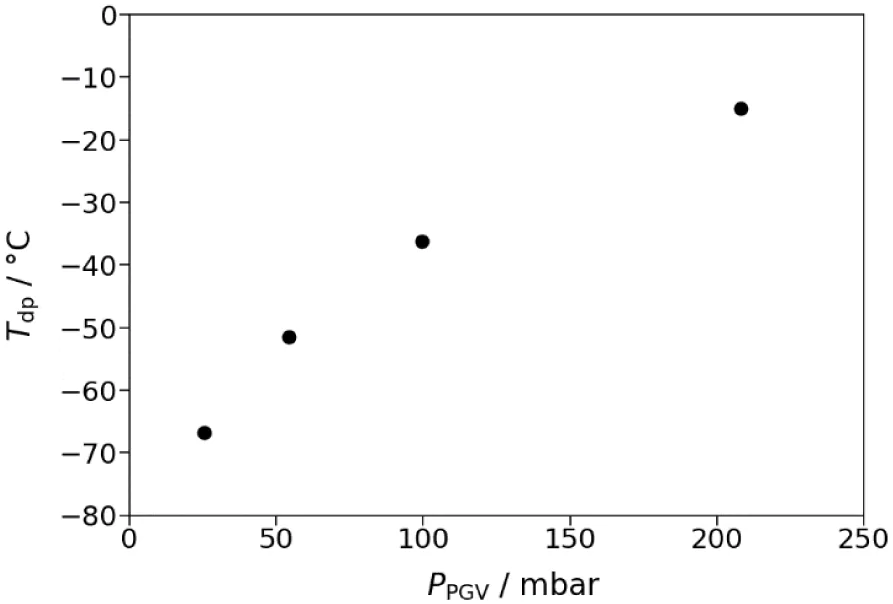
Experimental T_dp_ versus the regeneration pressure during PGV stage.

### Economic Assessment

At the laboratory scale, the operating conditions are optimized to deliver the highest recovery at the required hydrogen product purity. However, when scale-up is considered, this is not the best strategy, since the relevant objective is to minimize operating and investment costs (OPEX + CAPEX).

Table S6 reports the cost breakdowns for all experiments; note that this table displays simulated values with greater precision than the variables’ actual precision, to illustrate their trends. The optimum conditions and dependent variables values are shown in the text with the required number of digits.

### Technical Limitations of Scale-Up

As briefly mentioned in the *Experimental section*, the assumption that the pressure drop and concentration profiles during adsorption remain unchanged may not be entirely realistic. For example, setting the equalization and blowdown steps to 1 s, as considered for the cycle presented in Table 3 for small-scale columns, may induce fluidization and intense pressure fluctuations, leading to significant mechanical stresses and the erosion of the adsorbent. To mitigate these effects, the minimum depressurization time was estimated using the Wen and Yu correlation,^53^ which relates the Reynolds number (Re, eq 19) to the Archimedes number (Ar, eq 20) to determine the minimum fluidization velocity (*U*_mf_) for spherical particles:

19 $$Re_{\mathrm{mf}}=\frac{D_{p}U_{\mathrm{mf}}\rho_{g}}{\mu \left(1-\epsilon \right)}=\sqrt{33.7^{2}+0.0408\times \mathrm{Ar}}-33.7$$

20 $$Ar=\frac{d_{p}^{3}\rho_{g}\left(\rho_{p}-\rho_{g}\right)g}{\mu^{2}}$$

where *ρ* is the specific mass of H_2_, *μ* is the dynamic viscosity of H_2_, *ϵ* is the bed porosity (assumed 0.4), *g* is the gravitational acceleration, and subscripts p and g refer to the bead particles and the carrier gas (H_2_), respectively. The depressurization velocity was then set to 0.5 *U*_mf_, ensuring operation below the fluidization threshold and thereby preventing particle mobility and erosion for imperfectly packed beds under operating conditions of 7.8 bar and 28.5 °C.

At the highest operating pressure, 7.8 bar, is when hydrogen is more viscous (9.0 μPa·s) and denser (0.48 kg·m^–3^), resulting in the lowest 0.5 *U*_mf_ (0.47 m·s^–1^). At constant 0.5 *U*_mf_ and assuming ideal gas behavior, the minimum depressurization time to go from 7.8 bar to 25 mbar rounds to 40 s in the industrial-scale adsorption unit. In a cycle time spanning several hours, 40 s is a negligible fraction of the time it takes to complete regeneration. In the Supporting Information, the reader can find the algorithm of calculation of the depressurization time.

Admittedly, working with bead sizes of 0.84 mm at large scales could impact the pressure drop during the adsorption stage. A mass feed flow rate of 300 kg·h^–1^ corresponds to a superficial velocity of 0.354 m·s^–1^ at 7.8 bar for columns with dimensions *D*_i_ = 0.346 m and *H*_v_ = 3.46 m (Table S3). The Ergun equation (eq 21) allows computing the pressure drop:

21 $$\frac{\Delta P}{H_{b}}=\frac{150\mu (1-\epsilon {)}^{2}}{d_{p}^{2}\epsilon^{3}}U_{\mathrm{feed}}+\frac{1.75\rho \left(1-\epsilon \right)}{d_{p}\epsilon^{3}}U_{\mathrm{feed}}^{2}$$

where Δ*P* is the pressure drop inside the column and *U*_feed_ is the superficial velocity of the feed flow rate. Four bead sizes were evaluated, with diameters ranging from the laboratory-scale value of 0.84 mm up to 4.00 mm. Table 10 shows that the smallest beads (0.84 mm) produce a significant pressure drop of 0.172 bar. These observations suggest potential benefits of using larger beads in adsorption systems. Nevertheless, increasing bead diameter reduces the surface-area-to-volume ratio, thereby increasing external mass-transfer resistance, potentially broadening the concentration front.^30^

**Table 10:** Bead Size vs Pressure Drop for the Industrial-Scale Column

Bead size/mm	0.84	1.41	3.36	4.00
Δ*P*/bar	0.172	0.071	0.018	0.014
Δ*P*/%	2.20	0.91	0.24	0.18

### OPEX Costs

Assuming precompressed hydrogen is provided by the electrolyzer,^54^ the main OPEX is associated with the vacuum pump, estimated at 9138–9210 €·year^–1^ for *θ*_closed_ = 0. Figure 20 shows the OPEX normalized to annual hydrogen production (specific OPEX). For example, hydrogen production in the first experiment is given by 300 kg·h^–1^ × 8000 h × 98.98% recovery. As expected, experiments with higher vacuum pressures (vac1, vac2, vac3) require less powerful vacuum pumps and thus cost less.

**Figure 20:**
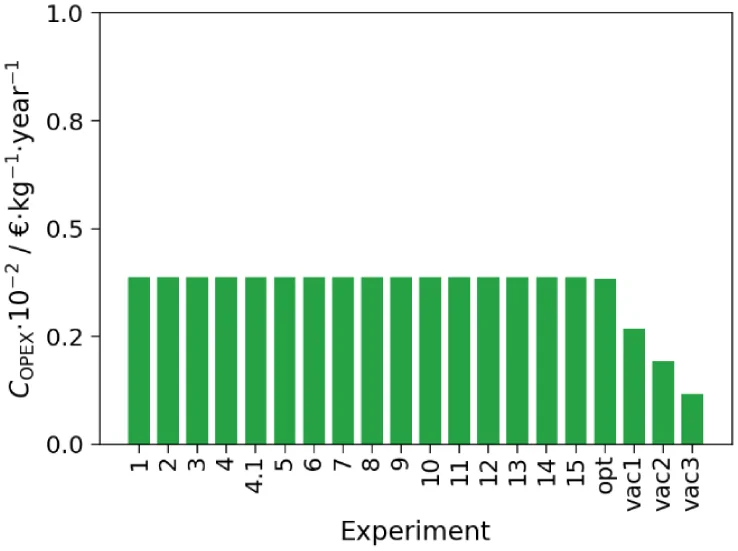
Operating expenditure costs per produced mass of H_2_ of each experiment considering an idealized 300 kg·h^–1^ of H_2_ VPSA unit.

To assess the need for a vacuum pump, it is essential to balance the amount of hydrogen available for purging if no vacuum pump is acquired. Operating a vacuum pump costs 9138 €·year^–1^ in the most favorable scenario, and the hydrogen selling price is 6.17 €·kg^–1^ as of November 2025.^55^ Thus, by replacing pumping operational costs with additional hydrogen consumption, approximately 1.48 tonne·year^–1^ of hydrogen could instead be employed as purge gas. Annually, 2.4 ktonne of hydrogen can be dried by the envisioned VPSA (300 kg·h^–1^). From this total, 1% is used for purge (24 tonne·year^–1^). The 1.48 tonne·year^–1^ of excess H_2_ traded by the vacuum pump accounts for 0.06% of the total available feed for additional purging, which is most likely not enough to bring *T*_dp_ from the presumable −9.62 °C obtained in the screening experiments (cycle #1) with 1% purging gas only, down to −65.5 °C.

The operating costs are related to the energy consumption of 0.038 kWh·kg^–1^ of H_2_, which contains some degree of uncertainty, derived from the power consumption correlations used. The achieved value is 1 order of magnitude lower than the value reported by Ligen et al. of 0.5 kWh·kg^–1^ of H_2_.^34^ Compared to the simulated result obtained by Tjarks et al.,^35^ it is over a 75% reduction from their reported 0.167 kWh·kg^–1^ of H_2_ at 10 bar in a TSA.

### CAPEX Costs

Capital costs comprise pressure vessels, adsorbent inventory, and the vacuum pump. Figure 21 shows the CAPEX per unit mass of hydrogen produced, based on a 5-year amortization period used in the ROI analysis. The cost term *C*_*v*_ aggregates the material costs for the three pressure vessels and the associated forging/welding costs. Material cost is a function of the wall thickness required to withstand the design pressure, which determines the steel volume; the wall thickness (*ε*) was calculated from eq S6. The calculated minimum thickness is always smaller than the commercial minimum specified by ASME Schedule 5 S (dimensions in Table 11). Adopting Schedule 5 S therefore increases vessel mass relative to the theoretical minimum but yields a more realistic cost basis.

**Figure 21:**
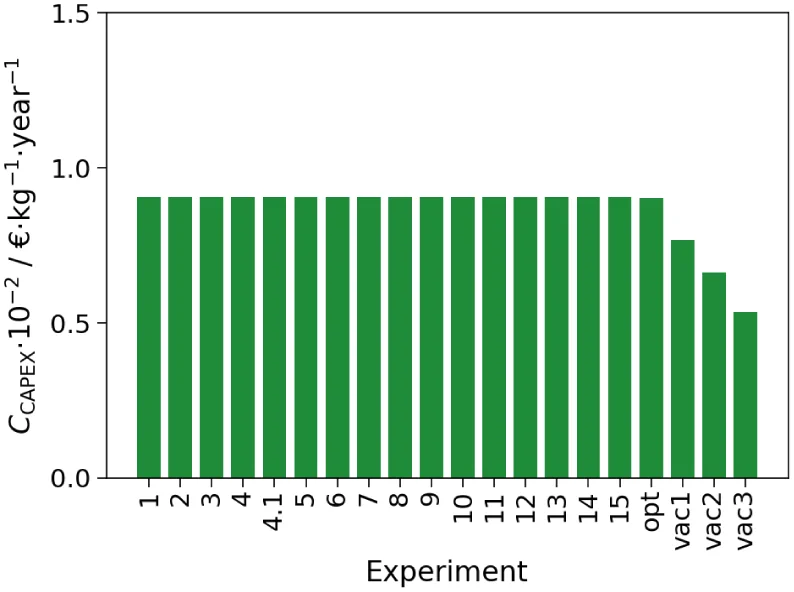
Capital expenditure costs per produced mass of H_2_ of each experiment considering an idealized 300 kg·h^–1^ of H_2_ VPSA unit.

**Table 11:** ASME Schedule 5 S Dimensions for the CAPEX Calculation of Pressured Vessels

Schedule 5 S dimensions	
*ε*/mm	4.19
*D*_0_/m	0.41
*H*_v_/m	4.10
*V*/m^3^	0.51
*m*_ads_/kg	399

A fabrication rate of 2.125 €·kg^–1^ of steel was applied for forging and welding.^56^ The adsorbent cost was updated to reflect the revised internal volume of the vessels, using a bulk packing density of 790 kg·m^–3^ determined from the laboratory setup.

### Revenue Modeling

Because the purge flow rate and operating pressure are fixed, the energy consumption of the vacuum pump remains nearly constant. As a result, cash flow is mainly governed by the recovered purified hydrogen flow rate stream. By fixing the dew point at *T*_dp_ = −67.5 °C, eqs 17 and 18 can be recast into eqs 22 and 23 for direct use in the revenue model.

22 $$P_{\mathrm{AD}}=2.781+2.091\times t_{\mathrm{AD}}-0.130\times t_{\mathrm{AD}}^{2}$$

23 $$R_{\mathrm{rec}}=98.964+0.0131\times t_{\mathrm{AD}}-0.00254\times t_{\mathrm{AD}}^{2}$$

The quadratic eqs 23 yields the modeled optimum given that the OPEX associated with each *t*_AD_ (and, consequently, each *P*_AD_) is invariant, as discussed previously. Figure 22a identifies the optimal operating conditions, *t*_AD_ = 2.6 h and *P*_AD_ = 7.34 bar, at which *R*_rec_ the highest (98.98%), which also corresponds to the lowest specific OPEX cost: 0.0039 €·kg^–1^·year^–1^; *cf*. Figure 22b–a negligible increase to the hydrogen cost base of 6.17 €·kg^–1^. The results indicate that the specific OPEX cost exhibits low variation across the *t*_AD_ value range, being primarily governed by the purge flow rates and regeneration pressure.

**Figure 22:**
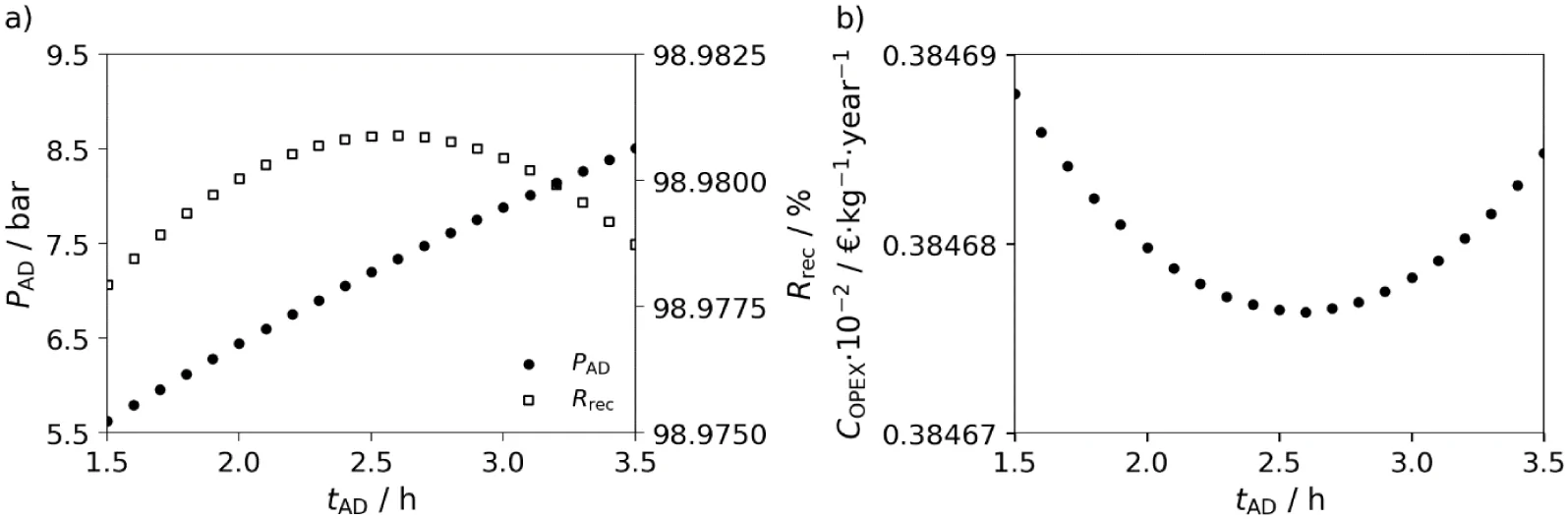
a) Variation of P_AD_ and R_rec_ as a function of t_AD_. b) C_OPEX_ as a function of t_AD_.

The levelized cost of hydrogen drying is the specific price at which the ROI reaches breakeven at a predefined maturity period, after which both CAPEX and incurred OPEX are fully amortized. Assuming a maturity period of five years, the principal loan corresponds to a CAPEX of 107.2 × 10^3^ € plus a cumulative OPEX of 45.7 × 10^3^ € under optimal operating conditions, resulting in a total principal of 152.8 × 10^3^ €. Applying an interest rate of 5.28% increases the total debt to 197.7 × 10^3^ €. The minimum LCOHD required to repay this total debt is therefore 0.017 €·kg^–1^·year^–1^, which represents only a marginal contribution to the final hydrogen selling price. In contrast, if a compressor is coupled downstream to the VPSA to output hydrogen at 700 bar for storage in high-pressure vessels, the estimation of the LCOHD would escalate to 1.98 €·kg^–1^ (*cf*. Supporting Information for calculation details), in accordance with the literature.^57^

Electricity prices vary geographically and therefore directly affect the cost of hydrogen drying. Table 12 presents the hydrogen drying cost, considering only operating expenses (*C*_OPEX_), and the cost including return on investment (*C*_LCOHD_), which incorporates both CAPEX and OPEX, under optimal operating conditions of 2.6 h and 7.34 bar for four electricity price scenarios. As predicted by the linear relationship given in eq S1, the *C*_OPEX_ increases proportionally with the electricity price.

**Table 12:** Influence of Electricity Price on OPEX and LCOHD

Electricity price/€·kg^–1^	OPEX/€·year^–1^	C_OPEX_/€·kg^–1^·year^–1^	C_LCOHD_/€·kg^–1^·year^–1^
0.05	4569	0.0019	0.014
0.10	9138	0.0038	0.017
0.20	18276	0.0077	0.022
0.30	27414	0.0115	0.027

## Conclusion

This work studies the use of a vacuum–pressure swing adsorption (VPSA) process for drying humid hydrogen to meet the specifications required by ISO 14687-2: dew point below −65.5 °C, using activated alumina as the desiccant.

Water vapor adsorption isotherms and breakthrough curves were obtained for commercial activated alumina from Induceramic. The adsorption of water vapor displays a type IV isotherm with a load capacity for 80% of relative humidity of *ca*. 10 mol·kg^–1^ and H_2_ capacity of 0.02 mol·kg^–1^ at 8 bar. Breakthrough experiments suggest that bed efficiency increases with pressure, due to slower movement of the concentration front and lower room temperature resulting from reduced absolute humidity load.

VPSA screening tests showed a synergistic effect between hydrogen purge gas and reduced-pressure regeneration, removing water more effectively than either process alone. This configuration was implemented in a three-bed laboratory VPSA, shifted by 120°, and a Box–Behnken design (BBD) was used to fit an empirical quadratic model. The selected operating variables were the adsorption time (*t*_AD_), the adsorption pressure (*P*_AD_), and a time ratio where regenerating and backfilling beds when closed to product hydrogen (θ_closed_); the stream leaving the column goes exclusively to the product exit. The operating conditions that maximize the recovery ratio (*R*_rec_) for the set dew point temperature (*T*_dp_) of the dried hydrogen stream are a time of adsorption (*t*_AD_) of 2.95 h, adsorption pressure (*P*_AD_) of 7.8 bar and a regeneration pressure (*P*_PGV_) of 25 mbar; the obtained *R*_rec_ was 98.98% and the *T*_dp_ was −66.76 °C. The fitted model forecast a *R*_rec_ of 98.98% and a *T*_dp_ of −67.36 °C.

For a hypothetical VPSA unit processing 300 kg·h^–1^ of H_2_, the process economics were found to be largely insensitive to the investigated range of cycle times and operating pressures, due to the limited variation in hydrogen recovery under constant regeneration conditions: 1% P/F and 25 mbar. The minimum hydrogen purification cost was estimated at 0.0039 €·kg^–1^, considering OPEX only, increasing to 0.017 €·kg^–1^ when including ROI over five years at a 5.28% interest rate, assuming a debt corresponding to CAPEX plus five years of OPEX.

## Supplementary Material



## Notes

Measurement of a single isotherm point; laboratory VPSA setup; economic analysis; cost of compressing hydrogen; operating conditions and parameters for the experimental section; breakthrough experimental conditions and results; algorithm for the calculation of the depressurization time in the large-scale unit; VPSA experimental conditions and results (PDF)

## Abbreviations

AA: Activated alumina
AD: Adsorption stage
BBD: Box-Behnken design
BD: Blowdown stage
BF: Backfill stage
CAPEX: Initial capital expenditures
CEPCI: Chemical engineering plant cost index
DoE: Design of experiments
DPE: Depressurization pressure equalization stage
EV: Evacuation stage
IDLE: Idle stage
ISO: International Organization for Standardization
LCOHD: Levelized cost of hydrogen drying
MTZ: Mass transfer zone
OPEX: Operational expenditure
P/F: Purge-to-feed ratio
PGV: Purge under vacuum stage
PPE: Pressurization pressure equalization stage
PSA: Pressure swing adsorption
RMSE: Root mean squared error
ROI: Return-on-investment
RSM: Response surface methodology
SS: Stainless steel
TSA: Temperature swing adsorption
VPSA: Vacuum-pressure swing adsorption

## Nomenclature

*b*: Equilibrium constant (bar^–1^)
*C*: Cost (€ or €·year^–1^ or €·kg^–1^ or €·kWh^–1^)
*D*: Diameter (m)
*f*: Heterogeneity factor (-)
*F*: Volumetric flow rate (L_N_·s^–1^)
*H*: Height (m)
*k*: Specific heat ratio of hydrogen (-)
*K*: Adjusting factor for C_CAPEX_ (-)
*m*: Mass (kg)
*ṅ*: Molar flow rate (mol·s^–1^)
*P*: Pressure (bar)
*q*: Adsorption capacity (mol·kg^–1^)
*Q*: Adsorption heat (J·mol^–1^)
*R*: Ratio (%)
R: Ideal gas constant (J·K^–1^·mol^–1^)
*S*: Maximum allowable stress (bar)
*t*: Time (s)
*T*: Temperature (K)
*U*: Velocity (m·s^–1^)
*V*: Volume (m^3^)
*W*: Water vapor concentration (mol·L_N_^–1^)
*y*: Volumetric fraction (-)

## Greek letters

α: Heterogeneity constant (-)
β: RSM quadratic model coefficients (dependent on input and response variable)
*ε*: Thickness (mm)
ϵ: Porosity (-)
η: Efficiency (-)
θ: Time fraction (-)
ρ: Density (kg·m^–3^)
Φ: Power (kW)
Ψ: Productivity (kg_H_2__·kg_ads_^–1^)

## Subscripts

ads: Adsorbent
b: Breakpoint
bed: Packed bed
c: Compressor
dp: Dew point
e: Electricity
feed: Feed stream
i: Internal or numbering integer
ly: Labor year
mf: Minimum fluidization
prod: Product stream
rec: Recovery
s: Saturation
v: Vessel
vp: Vacuum pump
w: Welding

## Superscripts

0: Reference conditions
^: Estimated variable
