# Supplementary material for: High-Performing Vacuum-Pressure Swing Adsorption Unit for Drying Hydrogen from Water Electrolysis
Source: Ind Eng Chem Res. 2026 Aug 4;65(32):17315–32. doi: 10.1021/acs.iecr.6c00750 (PMC13495766; doi:10.1021/acs.iecr.6c00750)
Supplement: Supplementary file 1 [file ie6c00750_si_001.pdf]

High-performing Vacuum-Pressure Swing Adsorption unit for drying hydrogen from water electrolysis

Tiago M. R. Santos(a,b,c), José M. Sousa(a,b,d), Frederico Relvas(c), Adélio Mendes(a,b)*

(a) LEPABE - Laboratory for Process Engineering, Environment, Biotechnology and Energy, Faculty of Engineering, University of Porto, Rua Dr. Roberto Frias, 4200-465 Porto, Portugal

(b) ALiCE – Associate Laboratory in Chemical Engineering, Faculty of Engineering, University of Porto, Rua Dr. Roberto Frias, 4200-465 Porto, Portugal

(c) Amnis Pura, Parque Empresarial Laborim, 100, Armazém C3, 4430-246 Vila Nova de Gaia, Portugal

(d) Departamento de Química – Escola de Ciências da Vida e do Ambiente, Universidade de Trás-os-Montes e Alto Douro, 5001-911 Vila-Real, Portugal

* Corresponding author: mendes@fe.up.pt

Supplementary information to the main manuscript can be found here for the Experimental section and Results and discussion.

Measurement of a single isotherm point

A single adsorption equilibrium point was determined using a volumetric procedure modified from Santos et. al. [1] with an additional tank TW, containing water in vapor-liquid equilibrium shown in Figure S1. Initially, tanks TL and TS were at pressure equilibrium, after which TS was isolated by closing valve VS. Water vapor from tank TW, maintained at vapor-liquid equilibrium, was introduced into TL through valve VW until the desired pressure was reached. After pressure stabilization in TL, valve VS was opened, allowing vapor to expand into TS, which contained the adsorbent sample. Adsorption proceeded until a new equilibrium pressure was established. The amount adsorbed was calculated from the known volumes of tanks TL and TS, the sample mass, the initial pressure in TL, and the final equilibrium pressure of the system allows a single adsorption equilibrium data point to be determined. To determine the hydrogen isotherm, the same procedure should be applied except that VL is used instead of VW.

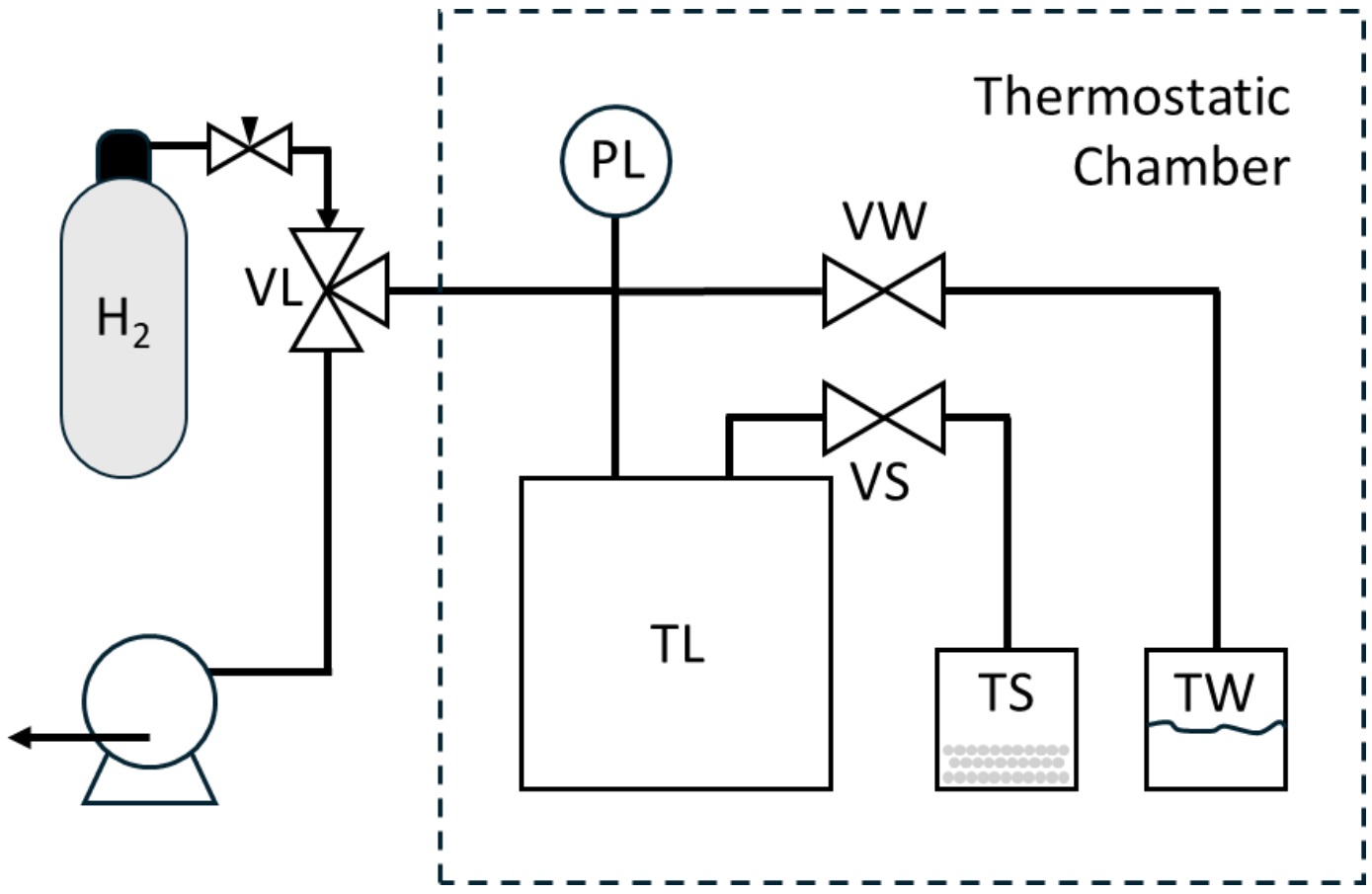

Figure S1 - Sketch of the setup used to obtain the adsorption Isotherms. NV: Needle valve; 3WV: Three-way valve; VW: Cut valve; VS: Cut valve; TL: Loading tank; TS: Sample tank; TW: Water tank; VP: Vacuum pump; P: Pressure transducer.

Laboratory VPSA setup

The setup represented in Figure 3 of the main text includes a thermostatic water column (length: 60 cm, internal diameter: 8.5 cm, thickness: 0.5 cm, and internal volume: 10.6 dm^3) to bubble and humidify the hydrogen feed stream at the required relative humidity. The operating pressure was set using an in-line electrical pressure controller (Bronkhorst P-702CV), while a rotary vane vacuum pump (VacuuBrand RZ 2.5) was employed to establish the vacuum level. The RH of the inlet stream was controlled by adjusting the thermostatic water column temperature and the VPSA operating room temperature (28.5 °C) to obtain a hydrogen feed RH of 80 %. The outlet T_{dp} was continuously monitored using a dew point meter (CS-Instruments FA500). The operating pressure (Druck PMP) of the columns was continuously read.

System control and data acquisition were carried out via a computer running an in-house LabView program. A PLC (Omron CJ2M) operated the solenoid valves (SCG325 above and SCE370 under the packed beds), while two acquisition cards (NI CB-68LP and Labjack U12) read and wrote the signals from all sensors and controllers. Each packed bed had an internal volume of 7.8 cm^3 and a length of 10 cm. The VPSA unit was assembled using Swagelok connectors, valves, and 1/8" and 1/4" stainless steel tubing.

Economic analysis

The system boundary comprised a vacuum pump, pressure vessels and the adsorbent inventory. OPEX is driven primarily by the electrical energy for the vacuum pump during a labor year as described by Eq. (S1):

$$C_{OPEX} = C_e \times \Phi_{vp} \times t_{ly} \tag{S1}$$

where C_{OPEX} is the OPEX yearly cost, C_e is the cost of electricity, Φ_{vp} is the power requirement for the vacuum pump and t_{ly} is the number of seconds in a labor year of 8000 h. C_e is the electricity cost, assumed to be 0.10 €·kWh^{-1}. Φ_{vp} was calculated from the pressure ratios across the high- and low-pressure sides using Eq. (S2) [2]:

$$\Phi_{vp} = \frac{\dot{n}\,\mathcal{R}\,T}{1000 \times \eta_p} \frac{k}{k\text{-}1} \left[\left(\frac{P_1}{P_2}\right)^{\frac{k\text{-}1}{k}} - 1 \right] \tag{S2}$$

where $\dot{n}$ is the molar flowrate, η_p is the power efficiency, k is the hydrogen specific heat ratio, P_1 is the high-pressure side, and P_2 is the low-pressure side. On the other hand, CAPEX sums the individual costs of the system boundary using Eq. (S3):

$$C_{CAPEX} = K \times \left[\left(C_{CEPCI_{2023}}/C_{CEPCI_{2001}} \times C_{vp}\right) + (C_v + C_{ads})\right] \tag{S3}$$

where K is an adjusting factor that considers additional direct and indirect costs of acquisition of ancillary items (piping, valves, electricals) and other services. CEPCI is the chemical engineering plant cost index that considers inflation-adjusted costs from the base year of 2001 to the latest year available of 2023 and was only applied to C_{vp} because it was obtained through correlations. C_{vp} is the cost of the vacuum pump, C_v is the cost of the adsorption vessels and C_{ads} is the cost of the adsorbent for the three vessels. C_{vp} was calculated following the empirical Eq. (S4) [3].

$$C_{vp} = 32500 \left(\frac{1.341\,\Phi_{vp}}{10}\right)^{0.5} \tag{S4}$$

The C_v for the three adsorption vessels was calculated according to Eq. (S5):

$$C_v = C_{SS} \times \rho_{SS} \times 3 \times \left(\pi H_v(\varepsilon D_o - \varepsilon^2) + (\pi/2) D_o^2 \varepsilon\right) \tag{S5}$$

where C_{SS} is the specific cost of stainless steel and ρ_{SS} is the specific mass of stainless steel. The adsorption vessels were assumed to be cylindrical steel tubes, closed at both ends, with a thickness determined by the maximum operating pressure of the system. As a first approach, the Barlow's formula was used to calculate the thickness requirements of the vessel walls under internal pressure represented in Eq. (S6) [4]:

$$\varepsilon = \frac{P_{AD} \times D_i}{29 \times S \times \eta_w - 0.0414 \times P_{AD}} \tag{S6}$$

where ε is the thickness of the walls, P_{AD} is the pressure during AD, D_i is the internal diameter of the cylindrical tubes, S is the maximum allowable stress of stainless steel, and η_w is the joint efficiency of welding.

The column dimensions were then obtained from the ASME B36.19M standard [5] by excess. For instance, the directly upscaled vessel from the laboratory unit to a unit producing 300 kg·h^{-1} of H_2 had a D_i diameter of 0.346 m and a height H_v of 3.46 m to respect the 1:10 ratio. An experiment operating at 8 bar would require a 1.18 mm-thick wall according to Eq. (22). The ASME dimension chosen by excess is a 16 in (0.406 m) outer diameter D_o vessel with a thickness of 4.19 mm, the thinnest available wall, resulting in a 0.398 m D_i vessel. This corresponds to a Schedule 5 S type.

To calculate the adsorbent cost, C_{ads}, its mass was obtained based on the volume of the pressure vessels and the bulk density of the activated alumina calculated from the laboratory setup in Eq. (S7).

$$C_{ads} = C_{AA} \times \rho_{AA} \times (3 / 4)\pi D_i^2 \quad \text{(S7)}$$

where C_{AA} is the specific cost of activated alumina, and ρ_{AA} is the bulk density of the commercial activated alumina. A return-on-investment (ROI) analysis was performed to determine the levelized cost of hydrogen drying (LCOHD) required to generate sufficient revenue to service a five-year loan covering the total C_{CAPEX} and five years of C_{OPEX}. The LCOHD (C_{LCOHD}) was calculated using a compound-interest debt formulation, as described in Eq. (S8).

$$C_{LCOHD} = \frac{(C_{CAPEX} + t \times C_{OPEX}) \times (1 + I)^t}{m_{H_2}} \quad \text{(S8)}$$

where t is the five-year loan time, I is the compound interest and m_{H_2} is the total mass of product hydrogen in a labor year. The interest rate is assumed to be 5.28 %, corresponding to the highest average rate for non-financial corporations over the past five years in Europe [6]. Table S3 summarizes the parameters used in the economic analysis equations.

Cost of compressing hydrogen

The OPEX and CAPEX for the compression of hydrogen are calculated as follows. Equation (S9) yields the power requirements of the compressor, denoted by the subscript c.

$$\Phi_c = \frac{\dot{n}\, R\, T}{1000 \times \eta_p} \frac{k}{k\text{-}1} \left[\left(\frac{P_1}{P_2} \right)^{\frac{k-1}{k}} - 1 \right] \quad \text{(S9)}$$

The cost of compressor is calculated by Eq. (S10) [3]:

$$C_c = 5840 \times \Phi_c^{0.82} \quad \text{(S10)}$$

OPEX and CAPEX are calculated with Eqs. (S11) and (S12), respectively:

$$C_{OPEX} = C_e \times \Phi_c \times t_{ly} \quad \text{(S11)}$$

$$C_{CAPEX} = K \times \left(C_{CEPCI_{2023}} / C_{CEPCI_{2001}} \times C_c \right) \quad \text{(S12)}$$

Operating conditions and parameters for experimental section

Table S1 summarizes the experimental testing conditions and the characteristic dimensions of the packed bed for breakthrough characterization in subsection Breakthrough characterization unit and methodology. Table S2 summarizes the static

operating conditions and packed bed characteristics for the VPSA experiments in subsection VPSA optimization cycle and evaluation. Table S3 summarizes the design parameters for the CAPEX and OPEX calculations in subsection Economic analysis.

Table S1-Experimental conditions and characteristics of the packed bed used in breakthrough experiments.

**Feed conditions**	
F_{feed} / $L_N \cdot min^{-1}$	1.5 and 0.75
T / °C	23 and 28.5
P / bar	4 and 8
RH %	50, 70 and 80
**Packed bed characteristics**	
H_{bed} / cm	10
D_i / cm	1.0
D_o / cm	1.3
V_{bed} / cm^3	7.85
m_{bed} / g	6.17
Thermocouple position relative to H_{bed}	0.1/0.5/0.9

Table S2-Static operating conditions and characteristics of the packed bed used in VPSA experiments.

**Operating conditions**	
F_{feed} / $L_N \cdot min^{-1}$	1.5
T / °C	28.5
RH	*ca*. 80 %
P/F	1 %
P_{EV} / bar	0.025
**Packed bed characteristics**	
m_{bed} / g	6.11
V_{bed} / cm^3	7.85
H_{bed} / cm	10

D_i / cm	1.0
D_o / cm	1.3

Table S3-Design parameters for the CAPEX and OPEX calculations.

**Pressure vessel parameters**	
D_i / m	0.346
H_v / m	3.460
V / m^3	0.326
**Vacuum pump parameters**	
η_P	0.60
k	1.41
t_{ly} / s	2.88×10^{7}
C_e / €·kJ^{-1}	2.778×10^{-5}
**Steel and welding parameters**	
η_w	0.85
S / bar	1378.95
ρ_{SS} / kg·m^{-3}	7850
C_{SS} / €·kg^{-1}	2.5
**Activated alumina parameters**	
ρ_{AA} / kg·m^{-3}	789.43
C_{AA} / €·kg^{-1}	5.0
**CAPEX parameters**	
K	1.2
$CEPCI_{2001}$	397
$CEPCI_{2023}$	797.9
**LCOHD parameters**	
I	0.0528
t / year	5

Breakthrough experimental conditions and results

Experimental parameters and results from subsection Breakthrough characterization are presented in Table S4-Parameters and results of breakthrough experiments for humid hydrogen.

Table S4-Parameters and results of breakthrough experiments for humid hydrogen.

	Experimental conditions						Results						
**Experiment**	**F_{feed} / $L_N \cdot min^{-1}$**	**P / bar**	**RH / %**	**T_{dp} / °C @ 1 bar**	**T / °C**	**Regeneration**	**T_{dp} min / °C**	**t_b / h (@ -65.5 °C)**	**t_s / h**	**q_b / $mol \cdot kg^{-1}$**	**q_s / $mol \cdot kg^{-1}$**	**H_{MTZ} / cm**	**η_{ads} (%)**
**BT1**	1.5	8	50	-10.7	28.5	overnight, vac, 1% P/F	-80.0	3.02	3.45	4.74	5.41	2.51	87.6
**BT2**	1.5	8	70	-6.9	28.5	overnight, vac, 1% P/F	-80.0	3.00	3.64	6.27	7.67	3.66	81.7
**BT3**	1.5	8	80	-5.4	28.5	overnight, vac, 1% P/F	-80.0	3.01	3.80	7.52	9.50	4.16	79.2
**BT4**	1.5	4	70	1.5	28.5	overnight, vac, 1% P/F	-80.0	1.20	1.65	5.26	7.47	5.49	72.5
**BT5**	1.5	4	35	-6.9	28.5	overnight, vac, 1% P/F	-80.0	1.57	1.98	3.46	4.34	4.08	79.6
**BT6**	1.5	8	70	-6.9	28.5	2.5 h, vac, 1% P/F	-72.2	-	3.08	-	6.78	-	-
**BT7**	1.5	8	70	-6.9	28.5	1.5 h, vac, 1% P/F	-48.9	-	2.14	-	4.70	-	-
**BT8**	1.5	8	70	-10.6	23	overnight, vac, 1% P/F	-80.0	4.38	5.24	6.50	7.77	3.27	83.7

Algorithm for the calculation of the depressurization time in the large-scale unit

In the Economic assessment subsection, the algorithm for the calculations of the depressurization time in the Technical limitations of scale-up using the column dimensions in Table S3 are shown in Figure S2. Table S5 shows the iterations at every 10 s including the 0th and the last iteration.

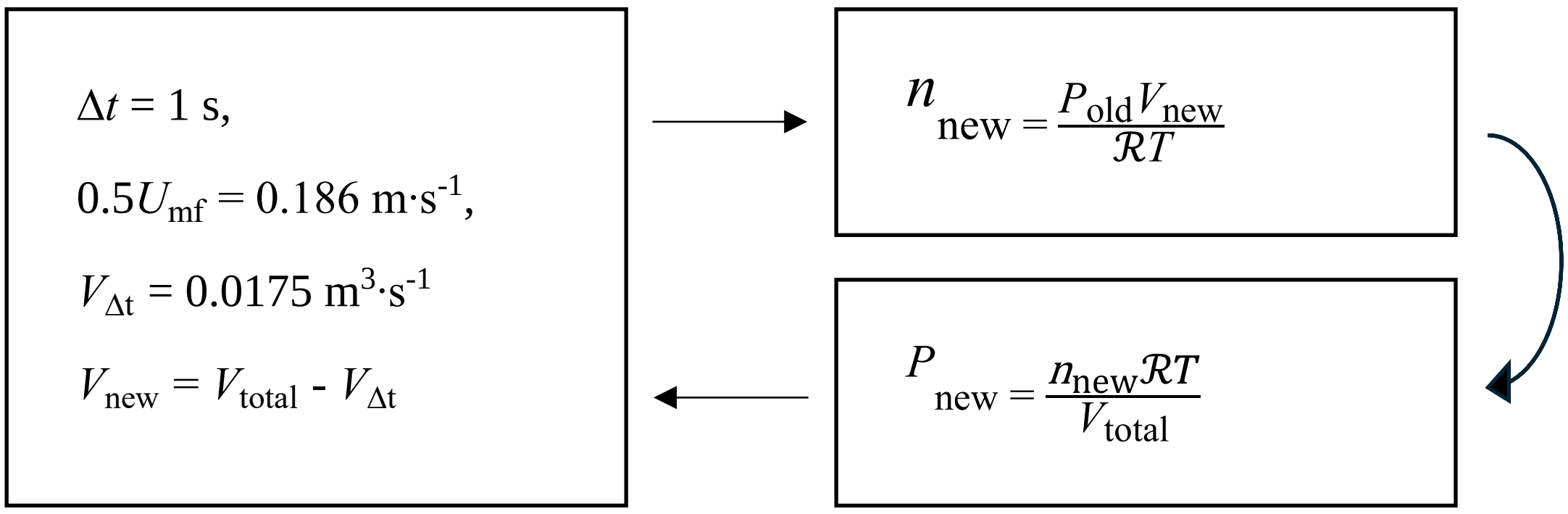

Figure S2-Algorithm for the calculation of iterated pressure during depressurization.

Table S5-Iterations for the calculation of depressurization time.

***t* / s**	**$V_{\Delta t}$ / $m^3 \cdot s^{-1}$**	**V_{new} / $m^3 \cdot s^{-1}$**	***n* / mol**	**P_{new} / bar**
0	-	0.325 (V_{total})	101.2	7.8 ($P_{initial}$)
10	0.0175	0.308	23.514	1.813
20	0.0175	0.308	5.465	0.421
30	0.0175	0.308	1.270	0.098
40	0.0175	0.308	0.295	0.023

VPSA experimental conditions and results

Table S6 compiles the experimental parameters and results for response surface methodology experiments, as well as the CAPEX and OPEX results for the scaled-up unit producing 300 kg·h^{-1} of H_2.

Table S6-Experimental conditions and results for the VPSA and expenditure calculations: i) DoE parameters; ii) Regeneration pressure and flowrate of experiments; iii) Experimental results; iv) estimated results based on BBD model; v) Power requirements and OPEX; vi) Cost of equipment and CAPEX.

	i			ii		iii			iv		v		vi				
Experiment	t_{AD} / h	P_{AD} / bar	ϑ_{closed}	P_{PGV} / bar	$\dot{n}_{PGV}$ / mol·s^{-1}	R_{rec} / %	T_{dp} / °C	Ψ / $kg_{H_2}\cdot kg_{ads}^{-1}$	$\hat{T}_{dp}$ / °C	$\hat{R}_{rec}$ / %	Φ_{vp} / kW	$C_{OPEX} \times 10^3$ / €·year^{-1}	ε^{γ} / mm	$C_{vp} \times 10^4$ / €	$C_v^3 \times 10^3$ / €	$C_{ads} \times 10^3$ / €	$C_{CAPEX} \times 10^5$ / €
1	3.50	8	0.0714	0.025	0.45	98.980	-62.86	4.62	-62.42	98.980	11.51	9.21	1.18	4.04	2.46	5.98	1.08
2	2.50	4	0	0.025	0.41	98.989	-35.70	3.31	-35.46	98.987	11.51	9.21	0.59	4.04	2.46	5.98	1.08
3	2.50	4	0.1428	0.025	0.48	98.990	-35.26	3.30	-35.46	98.987	11.51	9.21	0.59	4.04	2.46	5.98	1.08
4	1.50	8	0.0714	0.025	0.45	98.976	-80.00	1.98	-91.31	98.974	11.51	9.21	1.18	4.04	2.46	5.98	1.08
4.1[ʋ]	2.30	8	0.0714	0.025	0.45	98.979	-77.85	3.06	-78.50	98.979	11.51	9.21	1.18	4.04	2.46	5.98	1.08
5	2.50	8	0.1428	0.025	0.48	98.979	-76.39	3.30	-75.56	98.979	11.51	9.21	1.18	4.04	2.46	5.98	1.08
6	2.50	6	0.0714	0.025	0.45	98.981	-55.32	3.31	-55.51	98.983	11.51	9.21	0.89	4.04	2.46	5.98	1.08
7	1.50	6	0	0.025	0.41	98.978	-72.72	1.99	-71.26	98.977	11.51	9.21	0.89	4.04	2.46	5.98	1.08
8	1.50	4	0.0714	0.025	0.45	98.979	-49.47	1.99	-51.22	98.981	11.51	9.21	0.59	4.04	2.46	5.98	1.08
9	3.50	6	0.1428	0.025	0.48	98.984	-40.49	4.64	-42.37	98.983	11.51	9.21	0.89	4.04	2.46	5.98	1.08
10	3.50	6	0	0.025	0.41	98.984	-42.98	4.63	-42.37	98.983	11.51	9.21	0.89	4.04	2.46	5.98	1.08
11	1.50	6	0.1428	0.025	0.48	98.978	-71.56	1.99	-71.26	98.977	11.51	9.21	0.89	4.04	2.46	5.98	1.08
12	2.50	6	0.0714	0.025	0.45	98.981	-56.88	3.31	-55.51	98.983	11.51	9.21	0.89	4.04	2.46	5.98	1.08
13	3.50	4	0.0714	0.025	0.45	98.986	-23.28	4.64	-22.32	98.987	11.51	9.21	0.59	4.04	2.46	5.98	1.08
14	2.50	8	0	0.025	0.41	98.981	-74.69	3.31	-75.56	98.979	11.51	9.21	1.18	4.04	2.46	5.98	1.08
15	2.50	6	0.0714	0.025	0.45	98.981	-55.38	3.32	-55.51	98.983	11.51	9.21	0.89	4.04	2.46	5.98	1.08
opt	2.95	7.8	0	0.025	0.41	98.980	-66.76	3.92	-67.36	98.980	11.42	9.14	1.15	4.02	2.46	5.98	1.08
vac1	2.95	7.8	0	0.054	0.41	98.981	-51.53	3.90	-	-	7.98	6.39	1.15	3.36	2.46	5.98	9.12
vac2	2.95	7.8	0	0.099	0.41	98.983	-36.17	3.91	-	-	5.72	4.58	1.15	2.85	2.46	5.98	7.88
vac3	2.95	7.8	0	0.208	0.41	98.987	-15.04	3.91	-	-	3.46	2.77	1.15	2.21	2.46	5.98	6.35

ʋ - Same experiment as 4, but length of adsorption was increased so that contamination could be measured by the dew point meter.
ɣ - Considers Eq. (20) for the calculations.
ʒ - Considers the dimensions from the Schedule 5 S standard in Table 11.

References

(1) Santos, J. Study of New Adsorbents and Operation Cycles for Medical PSA Units. PhD., Faculdade de Engenharia da Universidade do Porto, 2005.

(2) Luyben, W. L. Capital Cost of Compressors for Conceptual Design. *Chemical Engineering and Processing - Process Intensification* **2018**, *126*, 206–209. https://doi.org/10.1016/j.cep.2018.01.020.

(3) Douglas, J. *Conceptual Design of Chemical Processes*; Clack, B., Bradley, W., Eds.; McGraw-Hill: Massachusetts, 1988.

(4) Megyesy, E. *Pressure Vessel Handbook*; Pressure vessel publishing, Inc: Tulsa, Oklahoma, 1997; Vol. 10.

(5) ASME. *B39.16M - Stainless Steel Pipe*; The American Society of Mechanical Engineers, 2018.

(6) Statista. *Average interest rate on loans to non-financial corporations in the euro area from January 2019 to June 2025*. European Central Bank. https://www.statista.com/statistics/941814/average-interest-rate-business-loans-in-euro-area/ (accessed 2026-02-02).
